# Treatment of Canine Atopic Dermatitis: Updated Guidelines From the International Committee on Allergic Diseases of Animals (ICADA)

**DOI:** 10.1111/vde.70128

**Published:** 2026-09-28

**Authors:** Ralf S. Mueller, Frane Banovic, Emmanuel Bensignor, Domenico Santoro, Tim Nuttall

**Affiliations:** ^1^ Small Animal Clinic LMU Munich Munich Germany; ^2^ Department of Small Animal Medicine and Surgery, College of Veterinary Medicine University of Georgia Athens Georgia USA; ^3^ Dermatoveto, Dermatology Referral Service (Paris) Rennes‐Cesson France; ^4^ Department of Small Animal Clinical Sciences, College of Veterinary Medicine University of Florida Gainesville Florida USA; ^5^ The Royal (Dick) School of Veterinary Studies and The Roslin Institute Midlothian UK

**Keywords:** allergy, dog, hypersensitivity, therapy

## Abstract

**Background:**

Canine atopic dermatitis (cAD) is a genetically, aetiologically, immunologically and clinically complex disease requiring both individualised immediate and long‐term management.

**Objectives:**

These updated guidelines from the International Committee on Allergic Diseases of Animals (ICADA) aim to substantially revise previous recommendations by organising treatment according to mechanism‐of‐action and by distinguishing two therapeutic phases: reactive therapy to resolve existing pruritus and skin lesions, followed by proactive therapy to maintain remission and reduce flares.

**Materials and Methods:**

The literature was reviewed and recommendations are based, where possible, on randomised controlled trials and systematic reviews, with evidence graded using the Strength of Recommendation Taxonomy. The guidelines review skin barrier care, antimicrobial and microbiome‐modulating interventions, avoidance of nonallergenic and allergenic flare factors, allergen‐specific immunotherapy, and anti‐inflammatory, antipruritic and immunomodulatory treatments.

**Results:**

Rapid‐acting broad‐spectrum drugs, particularly glucocorticoids, are recommended for reactive treatment. Janus Kinase (JAK) inhibitors and lokivetmab may be effective depending on lesion type, severity and inflammatory complexity. Secondary infections and flare factors must be addressed. Proactive management essential to prevent repeated cycles of inflammation may include skin barrier support, infection control, allergen avoidance, allergen immunotherapy, topical glucocorticoids, ciclosporin, tacrolimus, lokivetmab or JAK inhibitors, selected according to the patient's disease pattern and response.

**Conclusions and Clinical Relevance:**

A number of interventions are suitable for the treatment of cAD. Interventions are neither universally required, interchangeable nor effective in all cases. Treatment selection should take into account efficacy, safety, practicality, cost, the quality‐of‐life of both dog and owner, and the legal regulations applicable in the country of use.

## Abstract 2

**Background:** Canine atopic dermatitis (cAD) is a genetically, aetiologically, immunologically and clinically complex disease requiring both individualised immediate and long‐term management. **Objectives:** These updated guidelines from the International Committee on Allergic Diseases of Animals (ICADA) aim to substantially revise previous recommendations by organising treatment according to mechanism‐of‐action and by distinguishing two therapeutic phases: reactive therapy to resolve existing pruritus and skin lesions, followed by proactive therapy to maintain remission and reduce flares. **Conclusions and Clinical Relevance:** A number of interventions are suitable for the treatment of cAD. Interventions are neither universally required, interchangeable nor effective in all cases. Treatment selection should take into account efficacy, safety, practicality, cost, the quality‐of‐life of both dog and owner, and the legal regulations applicable in the country of use.

## 1 Background

In 2010, the International Task Force on Canine Atopic Dermatitis (ITFCAD), now the International Committee on Allergic Diseases of Animals (ICADA; www.icada.org) published the first comprehensive set of guidelines for the treatment of canine atopic dermatitis (cAD) [1]. ICADA undertook to keep these under review as new data on the disease and its treatment become available. The first update was published in 2015 [2]. The 2010 and 2015 guidelines are freely available online and in print, in English and multiple other languages. The present document represents a major rewrite of those guidelines, including published evidence until July 2026.

## 2 Principles Underlying the ICADA Guidelines and Treatment Recommendations

ICADA has established and followed a series of basic principles in writing these guidelines and formulating the treatment recommendations. The aim is to ensure that the guidelines are, as far as possible, based on objective evidence, thereby minimising any bias from personal experience or other sources. Readers are encouraged to familiarise themselves with these principles.

1. The recommendations are based, wherever possible, on published evidence from randomised controlled trials (RCTs) and systematic reviews [3, 4, 5]. Other published evidence is considered where RCTs or systematic reviews are not available. Published abstracts may be considered if there is no alternative *and* there are sufficient data to evaluate the conclusions. Widespread consensus practice may be considered if there is no alternative *and* this can be evidenced (e.g., in textbooks and review articles). Unsubstantiated opinion is not otherwise considered.
2. In the 2015 guidelines, the Strength of Recommendation Taxonomy (SORT) [6] was used to indicate the strength of a recommendation based on the quality and quantity of evidence for an intervention. An A‐level recommendation is based on consistent and good‐quality patient‐orientated evidence such as systematic reviews, meta‐analyses and concordant results from more than one randomised controlled trial (RCT) (Strength of Recommendation A = SOR A); a B‐level recommendation is based on inconsistent or limited‐quality patient‐orientated evidence such as at least one randomised controlled trial (RCT); a C‐level recommendation is based on descriptive studies and a D‐level recommendation is based on expert consensus. We follow this taxonomy again and either recommend a treatment or recommend considering this treatment as a possible option for individual patients, or state that there is insufficient evidence to recommend this particular therapy for cAD. A recommendation against any one of the evaluated treatments was not warranted based on the available evidence. A summary of the recommendations is given in Table 1.
3. Practitioners must bear in mind that statistically significant changes in the outcome measures for a clinical trial or other study do not mean that the intervention will be effective in all patients, or that the owners will be satisfied with the outcome. Clinical trials usually report the mean or median outcome with some measure of the variance. An individual animal may respond more favourably or worse than those in the study. Moreover, clinical trials usually assess the efficacy of a single intervention while best clinical practice in cAD frequently involves combining multiple treatments. Clinical trials may therefore underestimate the additive or synergistic potential of an intervention in combination with other treatments. Finally, a treatment may not be tolerated by a dog and/or be unacceptable to or unfeasible for an owner to apply irrespective of its published efficacy.
4. We use the statement *there is a lack of, or insufficient evidence supporting the efficacy* of some interventions. This does not mean that the intervention will not be effective in a given patient, and rather that it has not been tested sufficiently to assess if it offers any benefit to the average atopic patient.
5. As in the 2010 and 2015 guidelines where recommendations for an intervention are supported by one or more trials with a specific product, it may not be possible to extrapolate the results to similar products. We mention the generic drug name followed by the brand and company. In all other cases, recommendations only provide generic drug names.
6. Importantly, a recommendation for a specific product does not imply endorsement of the product or its manufacturer by the ICADA. A recommendation means only that there is at least one clinical trial supporting the product's efficacy in canine AD, or in the absence of such data, that there is a consensus for recommending an intervention.
7. Some of the therapeutics discussed in these guidelines are not licensed for use in animals. Clinicians should therefore be aware of regulations regarding informed consent and other restrictions on the use of unlicensed (i.e., off‐label) medication in their region or country of practice.
8. Finally, ICADA does not imply that all of the interventions in these guidelines are recommended—or even needed—for each patient or that they should be used in the order presented. When evaluating these recommendations, clinicians must consider the benefits, adverse effects, practicability and cost of each intervention alone or in combination as they apply to each affected dog and its owners.

**TABLE 1:** Treatment options for canine atopic dermatitis: Summary of recommendations.

Therapy	Recommendation	Justification	Subgroup consideration	Monitoring
Topical therapy (Sections 4.1 and 4.2)	Although variable evidence exists, we suggest offering the option of using topical skin barrier support as an adjunct for proactive maintenance therapy.	The desirable effects (skin barrier support, pruritus and skin lesion reduction) may help as an adjunctive therapy in some dogs with cAD.	Dogs with mild cAD.	Only clinical monitoring is needed owing to rare and mild adverse effects.
Essential fatty acids (Section 4.3)	We suggest offering the option of administering essential fatty acids as adjunct therapy or in very mild cases of cAD.	There is a (limited) decrease in lesions and pruritus. In addition, there is a drug‐sparing effect with glucocorticoids, ciclosporin and oclacitinib.	Dogs with mild cAD and atopic dogs on systemic glucocorticoids, oclacitinib or ciclosporin.	Only clinical monitoring is needed owing to rare and mild gastrointestinal adverse effects.
Microbiome modulation (Section 5.2)	We suggest offering the option as treatment for acute flares or proactive maintenance therapy. Oral probiotics administered pre‐ and postnatally may delay the development of cAD.	The desirable effects (pruritus and reduction of clinical signs) must be weighed against potential inconsistent efficacy. A treatment trial in mild cases may be worthwhile.	Dogs with mild cAD.	Only clinical monitoring is needed owing to rare and mild adverse effects.
Allergen immunotherapy (Section 6.3)	We recommend it for all dogs with cAD caused by environmental allergens.	A decrease in the need for additional medications, pruritus or lesions is seen in the majority of patients.	All dogs with cAD caused by environmental allergens.	Only clinical monitoring is needed owing to rare and mild adverse effects.
Antihistamines (Section 7.1)	We suggest offering the option of prophylactic treatment with antihistamines for flare prevention.	The desirable effects (minor pruritus reduction, potential sedation benefit) have to be weighed against inconsistent efficacy and the need for daily long‐term dosing. A treatment trial for flare prevention in mild cases may be worthwhile.	Dogs with mild cAD.	Only clinical monitoring is needed owing to rare and mild adverse effects.
Topical glucocorticoids (Section 7.2)	We recommend topical glucocorticoids for localised pruritus and acute to chronic lesions (including ears and feet) in both reactive treatment of flares and proactive maintenance therapy. The frequency should be tapered to the lowest that maintains remission. Hydrocortisone aceponate is preferred for long‐term maintenance therapy owing to its better benefit/risk profile.	The clinical improvements in pruritus, acute lesions and chronic lesions are fast and persistent.	The short‐term safety profiles are very good. Adverse effects are more likely with traditional topical glucocorticoids than hydrocortisone aceponate or mometasone, particularly with long‐term treatment.	Clinical monitoring for systemic or local (at the application sites) signs or iatrogenic hypercortisolism; no specific testing is required.
Systemic glucocorticoids (Section 7.3)	We recommend oral glucocorticoids for the reactive treatment of flares. Use with caution as long‐term treatment owing to the common occurrence of adverse effects.	The desirable effects (reliable and rapid reduction of pruritus and skin lesions) must be weighed against potential adverse effects in some dogs.	Use with caution owing to the many possible adverse effects.	Only clinical monitoring is needed owing to the recommended short‐term administration and the expected clinical adverse effects (e.g., polyuria, polydipsia, polyphagia).
*JAK‐inhibitors*				
Oclacitinib (Section 7.4.1)	We suggest offering the option as treatment for acute flare and as proactive maintenance therapy.	The desirable effects (pruritus and clinical signs reduction) are fast and persistent. However, improvement must be weighed against the rare potential adverse effects and immunosuppression.	All dogs with cAD.	Periodic monitoring of complete blood counts and serum biochemical results of dogs on long‐term treatment is recommended.
Ilunocitinib (Section 7.4.2)	We suggest offering the option as treatment for acute flare and as proactive maintenance therapy.	The desirable effects (pruritus and clinical signs reduction) are fast and persistent. However, improvement must be weighed against potential adverse effects and immunosuppression.	All dogs with cAD.	Regular monitoring of complete blood counts and serum biochemical results of dogs is recommended.
Atinvicitinib (Section 7.4.3)	Assuming similar efficacy as the other JAK based on published abstracts, we suggest offering the option as treatment for acute flare and as proactive maintenance therapy.	Only few abstracts published and limited experience prevent a final statement, yet the desirable effects (pruritus and clinical signs reduction) seem to be fast and persistent. Improvement must be weighed against the potential adverse effects and immunosuppression.	All dogs with cAD.	Regular monitoring of complete blood counts and serum biochemical results of dogs is recommended.
Lokivetmab (Section 7.5)	We suggest offering the option as treatment for acute flare or as proactive maintenance therapy.	Pruritus reduction is fast and persistent. Clinical lesions seem to resolve a little slower and may need additional anti‐inflammatory therapy.	All dogs with cAD, although inflamed skin may need additional anti‐inflammatory therapy.	Clinical monitoring for systemic or local adverse effect (at the application sites) signs.
Ciclosporin (Section 7.6)	We suggest offering the option as treatment for proactive maintenance therapy. It is not indicated for acute flares.	The desirable effects (pruritus and clinical signs reduction) are slow, yet persistent. However, improvement must be weighed against potential adverse effects and immunosuppression.	All dogs with cAD.	Periodic monitoring of complete blood counts and serum biochemical results of dogs on long‐term treatment is not usually necessary in absence of systemic clinical signs.

Abbreviations: cAD, canine atopic dermatitis; JAK, Janus kinase.

## 3 Format of These Guidelines—Towards Revising the Treatment Strategy for Canine Atopic Dermatitis

Recent advances in our understanding of the pathogenesis and management of cAD suggest that the approach used in the previous guidelines is no longer optimal [7]. Those guidelines were divided into three sections with recommendations: (i) management of acute flares; (ii) treatment of chronic skin lesions; and (iii) interventions to prevent relapses. The 2015 guidelines included two scenarios that described typical cases of acute and chronic cAD to illustrate these approaches. The main factor used to recommend treatments for acute or chronic cAD was the time required to achieve the clinical benefit. For example, glucocorticoids and oclacitinib were recommended for both acute and chronic cAD, while ciclosporin was recommended only for chronic disease [2].

The genetic background of cAD is complex and variable, which may lead to differences in the clinical expression of the disease and the response to treatment. Its aetiology may involve skin barrier defects, aberrant skin inflammation, allergic responses to environmental and/or food allergens, and dysbiosis of the cutaneous microbiome. It is not clear how these pathways relate to each other or whether all of them are involved in the disease in each individual at each stage of its disease [8, 9, 10, 11, 12, 13, 14]. Unsurprisingly, therefore, there is breed, individual and temporal variation in the type, distribution, extent and severity of the clinical signs [15]. Consequently, atopic dogs may present with a combination of acute (e.g., erythema and excoriation) and chronic (e.g., lichenification and hyperpigmentation) skin lesions of varying extent and severity. Moreover, cAD is the most frequent primary factor in recurrent bacterial and yeast infections, otitis externa (OE) and chronic pododermatitis with interdigital furunculosis, which may need specific treatment in addition to that for the cAD. This heterogenous nature of cAD is not reflected in the earlier guidelines, which could confuse practitioners using these to make treatment decisions. Clinicians should consider ‘acute’, ‘chronic’, ‘mild’ and ‘severe’ stages of the disease, when deciding on the most appropriate treatment for their patient; it is possible for a narrow spectrum agent to be highly effective for severe pruritus and yet have little to no effect on chronic inflammation and otitis.

The pruritus and inflammation in cAD are the end result of a complex immunological cascade that varies at different stages of the condition as well as between breeds and individual dogs [7, 12, 13, 14, 16]. This was not considered in the earlier guidelines that emphasised the time to achieve a response over the precise mechanism‐of‐action (moA) and spectrum of activity. This could lead to inappropriate use with the risk of treatment failure and/or adverse effects. In particular, the previous guidelines did not emphasise the importance of long‐term proactive maintenance therapy to prevent the march to chronic changes that can be associated with intermittent therapy in reaction to flares.

These guidelines will therefore also briefly review the MoA and spectrum of anti‐inflammatory activity when making recommendations for each therapeutic intervention [7]. They will discuss recommendations for two distinct phases of treatment:

1. Phase 1 Reactive Therapy—the treatment of existing acute and/or chronic skin lesions and pruritus to resolve the clinical signs of the disease and induce clinical remission.
2. Phase 2 Proactive Therapy—subsequent long‐term regular therapy to maintain remission and minimise the number of flares.

These revised and updated guidelines will group therapeutic interventions by MoA (i.e., skin barrier care, antimicrobial and/or microbiome modulating therapy, allergen avoidance and allergen‐specific immunotherapy [AIT] and anti‐inflammatory/antipruritic therapy). After a brief summary of the MoA, each section will review and discuss the suitability of each intervention for Phase 1 Reactive and Phase 2 Proactive Therapy and will conclude with a brief recommendation. Finally, there will be a summary table of the evidence for each intervention (see Tables S1–S8) and a one‐page overall summary of the recommendations (Table 1).

## 4 Skin Barrier Care

### 4.1 Bathing With a Nonirritating Shampoo

#### 4.1.1 Mechanism‐of‐Action

A demulcent (e.g., urea, glycerine, propylene glycol) is a high molecular weight compound in aqueous solution that coats the skin surface, thus protecting the underlying cells, preventing transepidermal water loss (TEWL) and alleviating irritation. Linoleic acid is important for the barrier function of the stratum corneum (SC), particularly related to acylceramides that incorporate linoleic acid to form the corneocyte lipid envelope [17]. Colloidal oatmeal hydrates the SC hygroscopically by attracting and binding water passing through the epidermis [18].

#### 4.1.2 Evidence of Efficacy

Regular bathing with a mild nonirritating shampoo and lukewarm water is likely to increase skin hydration, have a direct soothing effect, remove surface allergens and debris, and inhibit potentially pathogenic bacteria and *Malassezia*. Small double‐blind RCTs have shown that weekly or more frequent bathing with an emollient shampoo can have a modest effect on cAD. In one study, twice‐weekly bathing with either a lactoferrin, chlorhexidine, piroctone olamine, chitosan, essential fatty acids and glycerine‐based shampoo (Dermatopic; Almapharm) or the placebo vehicle significantly reduced pruritus yet not lesion scores [19]. Weekly baths (10 min) with a lipid, complex sugars and antiseptic‐based shampoo (Allermyl; Virbac) halved pruritus scores within 24 h in four of 16 treated dogs [20]. The antipruritic effect was greater in a whirlpool bath than with conventional shampooing; the whirlpool used without shampoo and with water alone had similar efficacy in one of five dogs. Similar modest and short‐lived benefits were reported in other studies [21, 22]. A range of shampoos, foams and pads based around the plant‐derived ingredient Ophytrium has shown efficacy in reconstructed human and canine epidermal models as well as in atopic dogs (see Section 4.1 for more details). Some studies have shown that less frequent bathing in combination with other topical therapies is just as helpful as weekly bathing (see Section 4.1 for more details).

#### 4.1.3 Evidence of Safety

Shampoos may be drying and irritating and/or provoke contact reactions in individual patients. If necessary, clinicians should consider changing products or protocols and/or adding post‐bathing topical moisturisers containing ingredients such as lactic acid, propylene glycol or urea. The impact of frequent bathing on the reduction of efficacy of topical flea control products also should be considered.

#### 4.1.4 Recommendations

- Bathing once weekly (or less frequently in combination with other topical formulations) is a useful adjunct in the management of cAD (SOR A) [1, 2].
- This is likely to show most benefit in milder cases and when used regularly in proactive maintenance therapy (SOR D). However, intensive bathing may give rapid and short‐term relief from pruritus associated with an active flare and consequently be used as reactive therapy (SOR C).
- There is no evidence for the superiority of any particular shampoo or protocol, and the intensity and frequency of bathing may be the most important factor in relieving pruritus (SOR D).
- The type of shampoo should be tailored to each case: moisturising shampoos are likely to be the most soothing, yet anti‐seborrhoeic formulations may be more appropriate in dogs with seborrhoea and scaling (SOR C).
- Clinicians also should be prepared to change the topical products used if the state of the dog's skin and coat changes.

### 4.2 Topical Treatments Supporting the Skin Barrier

#### 4.2.1 Mechanism‐of‐Action

The results of measurement of skin barrier health and function through in vivo studies of topical nonmedicated products in dogs are controversial; therefore, many claims are based on in vitro studies or clinical improvements. The presumed MoA of topical nonmedicated products is based on the direct influence on skin barrier health by fatty acids, ceramides or humectants.

#### 4.2.2 Evidence of Efficacy

Less frequent bathing in combination with nonrinsing foams can be effective in pruritic dogs [23]. A multicentre, open‐label, uncontrolled study of ophytrium‐based products (Douxo S3 Calm; Ceva) in 34 atopic dogs showed improvement in most dogs with bathing on Day (D)1, followed by applications of a mousse every 2 to 3 days for 3 weeks (although concomitant systemic anti‐inflammatory/‐pruritic symptomatic medications were allowed) [23].

Another plant essential oil‐derived foam designed to improve skin barrier function (Atop7; Dermoscent) mildly improved pruritus and lesions after application twice weekly for 2 weeks [24]. Omega‐6 EFA‐ and essential plant oil‐containing topical spot‐on and spray formulations (Essential6 and Atop7; Dermoscent) had a modest effect on pruritus and clinical lesions in a small 8‐week open clinical trial in mildly atopic dogs [25], and similar findings were reported after treatment with the spot‐on formulation (Essential6; Dermoscent) in a small RCT [26]. However, using another spot‐on with lipids, ceramides and cholesterol (Allerderm; Virbac) twice‐weekly was no more effective than placebo for pruritus, lesions and measures of skin barrier function over 12 weeks of treatment [27]. In a further nonplacebo‐controlled study, IL‐31 monoclonal antibody therapy (Lokivetmab; Zoetis) in combination with bathing followed by a spot‐on containing topical essential fatty acids, ceramides and plant extracts (Atop7; Dermoscent) extended the time to a cAD flare compared to using the monoclonal antibody therapy alone [28] (Table S1).

#### 4.2.3 Evidence of Safety

In studies using topical treatments supporting the skin barrier, adverse effects were typically not observed in treated dogs [23, 25, 26, 27, 29].

#### 4.2.4 Recommendations

- There is variable‐quality evidence for using certain topical skin barrier support products as adjunct therapy in cAD (SOR B).
- These would be best used in combination with other therapies in the management of canine AD (SOR B).
- Topical skin barrier support products are likely to show most benefit in milder cAD cases and when used regularly in proactive maintenance therapy (SOR D).

### 4.3 Nutritional Supplements

#### 4.3.1 Mechanism‐of‐Action

Nutritional supplements include vitamins and minerals, essential fatty acids (EFAs) and probiotics (the latter are discussed in Section 4.2). EFAs may modulate the immune response in several ways. Linoleic acid plays an important role in the skin barrier. Furthermore, fatty acids have been shown to influence the T‐cell response in atopic dogs in vitro [30].

#### 4.3.2 Evidence of Efficacy

A recent review has summarised the evidence for nutritional supplements [31]. Oral intake of EFAs, especially those rich in omega‐6 EFAs, either as supplements or in enriched diets, can influence superficial skin lipid composition, reduce TEWL and improve the gloss and quality of the coat. However, these benefits, if any, may take months to become apparent [1, 2].

Three RCTs showed that oral EFA supplements significantly reduced the dose of prednisolone [32], ciclosporin [33] and oclacitinib [34] required to maintain clinical remission in atopic dogs. In one double‐blinded RCT, clinician and owner clinical scores improved significantly in dogs treated for 10 weeks with flax oil or eicosapentaenoic acid (EPA) and docosahexaenoic acid (DHA) in a commercial preparation (3VCaps; DVM Pharmaceuticals) compared to the placebo [35]. Another cross‐over RCT showed that an oral EFA supplement significantly reduced pruritus and lesion scores compared to a placebo, although the results have only been published in abstract form [36].

In general, EFA‐enriched diets provide higher amounts of EFA than oral supplements [1, 2]. In a single‐blinded cross‐over study, an EFA‐enriched diet (Dermatosis FP; Eukanuba) had a moderate effect on pruritus and lesion scores in cAD compared to a home‐cooked diet using the same main ingredients [37]. Another RCT showed improvement in lesion scores, pruritus and coat quality after 12 weeks of feeding with two high EFA diets (Veterinary HPM Dermatology support D1 [Virbac] and d/d [Hill's Pet Nutrition]) [38]. Dogs fed a novel therapeutic diet formulated for the management of cAD had a significant decrease in pruritus after 3 months and significantly decreased concurrent medication use after 6 months of feeding [39].

Palmitoylethanolamide (PEA) is an endogenous fatty acid amide related to endocannabinoids. Supplementation with an oral ultramicronised PEA formulation improved pruritus, lesion and quality‐of‐life (QoL) scores in an open clinical trial [40].

#### 4.3.3 Evidence of Safety

These supplements appear to be safe. Their MoAs make significant adverse effects unlikely, and none have been reported in the clinical trials. Potential concerns with EFA‐enriched diets and supplements, however, include weight gain and gastrointestinal upsets. Additionally, these diets may not be suitable for dogs with food‐associated cAD that are sensitised to one or more of their ingredients and for dogs that require a low‐fat diet (e.g., owing to pancreatitis).

#### 4.3.4 Recommendations

- There is limited evidence to recommend using these supplements or supplemented diets alone to manage cAD (SOR B).
- Essential fatty acids are safe and mildly effective for CAD (SOR A) and may be best used in combination with other therapeutics (SOR B).
- These supplements are not suitable for managing acute flares or chronic inflammation; they are likely to show most benefit in milder cases and when used regularly in proactive maintenance therapy (SOR D).
- There is no evidence for the superiority of any particular EFA or other supplement combination, dosage, ratio or formulation (including enriched diets) (SOR B). Moreover, whether or not similar effects would be seen with other supplements or enriched diets is unknown and care must be taken to not extrapolate the findings to other untested products (SOR D).

## 5 Antimicrobial and/or Microbiome Modulating Therapy

### 5.1 Antimicrobial Therapy

#### 5.1.1 Evidence of Efficacy

Bacteria (mainly *Staphylococcus pseudintermedius* ) and/or yeast (mainly *Malassezia* spp.) overgrowth or dysbiosis is common in cAD; these may evolve to recurrent superficial infections and otitis if the underlying cutaneous inflammation is not controlled. Such infections are generally associated with an exacerbation of the clinical signs of cAD and identified as perpetuating factors of pruritus and inflammation [41]. For these reasons, suspected bacteria and/or yeast infections should be confirmed via a cytological examination and properly treated. However, making antimicrobial treatment decisions based solely on cytological outcome and microbe numbers is inappropriate because other factors, such as microbial virulence and host response, play an important role in the genesis of clinical signs. This is specifically true for *Malassezia* dermatitis associated with yeast hypersensitivity reactions [42]. In this case, even a ‘low’ number of organisms can be associated with significant clinical signs. By contrast, a ‘high’ number of organisms might not cause any exacerbation of clinical signs if they are characterised by low pathogenicity and virulence with minimal activation of the immune response.

Nevertheless, the identification of a skin and/or ear infection as contributing factor of cAD is essential for appropriate management of the condition. In these cases, clinicians are encouraged to (i) properly identify skin and/or ear lesions (e.g., erythema, oedema, scaling, greasiness), (ii) cytologically confirm the presence of bacteria/yeast/inflammatory cells at these lesional sites, (iii) implement an appropriate antimicrobial regimen, (iv) document reduction/disappearance of skin lesions at the previous sites following antimicrobial interventions and (v) cytologically confirm the resolution of the infection following antimicrobial interventions [43].

Topical antiseptics should represent the first line of treatment in affected dogs [43]. The use of systemic antibiotics, at the proper dosage and length of administration, should be limited to cases of deep pyoderma and/or cases in which topical therapy is unsuccessful or contraindicated. The use of culture and susceptibility testing before systemic therapy also is highly recommended, particularly if antibiotics outside of the first‐choice group are prescribed. Similar to antibiotics, judicious use of systemic (e.g., ketoconazole, itraconazole, terbinafine) and/or topical antifungals to treat yeast infections is highly encouraged [44]. Such recommendations reflect the increased antimicrobial resistance seen in clinical practice for both bacteria and yeasts. Likewise, to avoid the increase in antimicrobial pressure, systemic or topical intermittent antimicrobial therapy (i.e., ‘pulse’ therapy) must be avoided. This latter statement also is based on the lack of evidence that long‐term regular use of topical antiseptics can reduce the recurrence of infections in dogs.

The most used antimicrobial formulations are solutions containing different concentrations of chlorhexidine with or without the addition of an antifungal (e.g., miconazole or ketoconazole). Shampoos containing benzoyl peroxide and ethyl lactate are still used, although the former may be contraindicated in atopic dogs because of the high drying effect associated with benzoic acid. In these cases, the use of a leave‐on conditioner to increase cutaneous hydration is essential. Very few studies have been published on the effectiveness of bleach and hypochlorous acid as treatment of canine pyoderma or bacterial otitis [45, 46, 47]. Furthermore, studies have confirmed the positive antimicrobial effect of natural plant‐based products as a valid alternative to classic antiseptics [47, 48, 49]. The higher rehydrating and soothing effect associated with such products is an additional benefit in atopic dogs.

In order to improve efficacy and antimicrobial stewardship, veterinary surgeons are advised to follow antimicrobial treatment guidelines established in their country of practice and/or in international consensus recommendations [43, 44, 50]. Clinicians should note that all recurrent skin and ear infections in dogs are secondary to underlying causes. The underlying cause must be identified and managed for a good long‐term outcome and repeated courses of antibiotic or antifungal treatment must be avoided.

#### 5.1.2 Evidence of Safety

These treatments are generally safe. The most frequent adverse effects are gastrointestinal upsets following systemic antimicrobial treatment, although clinicians should be aware of specific adverse effects associated with antimicrobials and be cautious in using them in at‐risk patients (e.g., hepatotoxicity and drug interactions with azole antifungals). Rare cutaneous and/or systemic adverse drug reactions are reported. As discussed above, shampoos may be drying and irritating and/or provoke contact reactions in individual patients.

#### 5.1.3 Recommendations

The importance of the role that infections play in the exacerbation of clinical signs of cAD in dogs has not changed from the earlier cAD treatment guidelines [1, 2]. However, since 2015, several studies have been published on the use of alternative options to treat superficial infection in atopic patients. Most of these studies were laboratory studies, yet some were followed by clinical trials. Additionally, consensus papers and new guidelines have been published to better guide clinicians in the identification, diagnosis and treatment of bacterial and yeast infections in companion animals. The central role that bacteria and yeast infections play in cAD is still very clear, and the treatment of such infections is a fundamental step in the treatment plan for atopic dogs, independently of the phase of the disease (proactive vs. reactive).

### 5.2 Microbiome Modulation

#### 5.2.1 Mechanism‐of‐Action

The interplay between microbes and host is essential for correct homeostasis. Likewise, the continuous crosstalk between cutaneous microbes and skin cells plays an essential role in the pathogenesis of cAD [11, 12]. Modulating the cutaneous microbiome to decrease the presence of pathogenic organisms (e.g., *S. pseudintermedius* ) and increase of beneficial organisms (e.g., lactobacilli and coagulase‐negative staphylococci) may be useful in the management of cAD. The mechanisms behind this modulation are not completely understood; however, a competitive role of beneficial bacteria along with the generation of more tolerogenic substances by host cells exposed to beneficial organisms has been demonstrated in vitro [51, 52].

#### 5.2.2 Evidence of Efficacy

Microbiome modulation may be an alternative to traditional antimicrobial therapy for cutaneous dysbiosis and to classic anti‐inflammatory medications to treat cAD in the future. However, at this time, there is insufficient evidence to support the beneficial effects of oral probiotics as treatment for cAD (Table S2). Pre‐ and postnatal exposure to the probiotic *Lactobacillus rhamnosus* strain GG has shown some possible lasting effect in reducing clinical signs following allergen challenges in dogs experimentally sensitised to house dust mites (HDM) [53, 54, 55]. Oral administration of *Lactobacillus paracasei* K71 (Hifu Sukoyaka Support; Kameda Seika) did not decrease pruritus or lesions compared to treatment with cetirizine, yet medication scores decreased significantly compared to the control group [56]. Oral heat‐killed *Escherichia coli* FK‐23 (Tsuyatto; NichiNichi Pharmaceutical) significantly improved skin lesions in atopic dogs compared to placebo treatment; however, pruritus and medication scores were not different between groups [57]. In a more recent open‐label clinical trial, topical use of a commercially available spray (Linkskin spray; Nextmune) composed of tyndallised lactobacilli ( *L. rhamnosus* strain GG and *Lactobacillus*), their culture media, vitamin D and polyphenols, showed significant amelioration of clinical signs in atopic dogs [58]. However, no changes in the cutaneous microbiome were detected after 4 weeks of daily applications. Very recent similar results, although preliminary, have been seen in an open‐label clinical trial with daily application of a spray solution containing live *Lactiplantibacillus plantarum* YUN‐V2.0 and *Lacticaseibacillus rhamnosus* YUN‐S1.0 in allergic dogs [59].

Although limited to human medicine [60, 61, 62], there is evidence supporting the potential use of cutaneous application of beneficial bacteria as an alternative treatment for cutaneous dysbiosis. The effects of topical antimicrobials on the cutaneous microbiome have not been fully studied in veterinary medicine. However, a very small clinical trial assessed the effect of a chlorhexidine/miconazole shampoo (Malaseb; Dermcare‐Vet Pty Ltd) applied twice‐weekly for 3 weeks [63]. At the end of the study, no changes in the microbiome were seen after 3 weeks of treatment and 4 weeks after discontinuation bathing when compared to healthy controls and over time.

#### 5.2.3 Evidence of Safety

These preparations appear to be safe, although there is a lack of large‐scale and/or long‐term data on safety and effects on the skin, gut and other microbiomes.

#### 5.2.4 Recommendations

- There is limited evidence to recommend using oral probiotics to manage/prevent cAD (SOR B).
- Products containing beneficial bacteria (live or dead) may have an effect on the cutaneous dysbiosis as reported in human medicine (SOR B). However, there is as yet limited to no evidence that the topical use of products containing tyndalised or live probiotics affects the cutaneous microbiome (SOR B).
- When used alone (SOR C) or in combination with other modalities (SOR D), topical products containing tyndallised or live probiotics are considered safe and effective to manage mild cases of cAD (SOR C).
- These supplements are not suitable for managing acute flares or chronic inflammation; they are likely to show most benefit in milder cases and when used regularly in proactive maintenance therapy (SOR D).

## 6 Avoidance of Nonallergenic Flares, Allergen Avoidance and Allergen‐Specific Immunotherapy

### 6.1 Avoidance of Nonallergenic Flare Factors

#### 6.1.1 Mechanism‐of‐Action

Avoidance and control measures for cAD aim to reduce exposure to nonallergenic and allergenic flare factors to keep the overall ‘trigger load’ below the threshold required to induce clinical flares. Nonallergenic factors in human AD include environmental (e.g., low humidity, clothing, detergents) and psychological triggers (e.g., stress); analogous factors may influence dogs, although evidence is limited.

#### 6.1.2 Evidence of Efficacy and Recommendations

In dogs, there is insufficient evidence to make general recommendations regarding the importance of environment, humidity, detergents and stress as flare factors. Owners are therefore encouraged to identify specific situations associated with worsening of their dog's condition and to avoid or modify them [1].

Mood‐modifying drugs and behaviour modification are appropriate where stress/anxiety is likely to be associated with flares of cAD (SOR D).

These approaches are not suitable for managing acute flares or chronic inflammation. They are most likely to be of benefit in avoiding flares when integrated into proactive maintenance therapy (SOR D).

### 6.2 Allergen Avoidance

#### 6.2.1 Mechanism‐of‐Action

Allergenic flare factors in cAD include environmental allergens (especially HDM and pollens), food allergens, and flea or other insect bites. Acute flares occur when hypersensitive dogs are exposed to sufficiently high allergen loads. Identifying and, where possible, eliminating contact with or ingestion of these allergens is important to prevent deterioration or recurrence of flares [1]. Control of fleas and other ectoparasites reduces exposure to flea salivary antigens, to which atopic dogs are predisposed to become hypersensitive with repeated bites [64]. Food restriction–provocation trials (‘elimination diets’) are used to identify food allergens that contribute to chronic, recurrent, year‐round signs.

Environmental aeroallergens, such as HDM, can induce flares in experimentally sensitised dogs [65, 66]. Environmental control measures, such as reducing HDM burden (e.g., acaricides, mattress covers, adjusting relative humidity, washing and vacuuming), aim to decrease allergen exposure in sensitised dogs.

#### 6.2.2 Evidence of Efficacy

Flea control has become much easier and more effective in the last few decades [67]. In acute cAD exacerbations, particularly in flea‐endemic areas, practitioners should ascertain whether fleas might have contributed to the worsening of signs. Atopic status predisposes dogs to hypersensitivity to flea salivary antigens with repeated exposure [64].

Food allergens can precipitate flares of cAD in sensitised dogs, which typically show chronic, recurrent, year‐round signs. However, diagnostic testing for food allergens has proven unreliable [68]. Food‐specific immunoglobulin (Ig)E/IgG serology offered by two unidentified commercial laboratories did not differentiate dogs with cutaneous adverse food reactions from those with nonfood‐induced diseases [69]. Consequently, one or more restriction–provocation dietary trials must be performed in all dogs with perennial (nonseasonal) cAD to determine whether food allergens contribute to clinical signs [68]. Normally, dietary trials should be carried out for 6–10 weeks using commercial or homemade diets containing a low number of novel and/or hydrolysed ingredients [70]. If a dietary trial failed early in the course of the disease, this aspect should be reconsidered in case of disease flares, especially if anti‐inflammatory therapy is not or no longer effective. Atopic dogs may acquire new hypersensitivities, and the development of a novel food allergy could be the cause of cAD exacerbation. More details about elimination diet trials can be found elsewhere [71]. In a small Japanese study, most dogs with signs of allergic skin disease, negative IgE serology to environmental allergens and a positive lymphocyte proliferation test to food allergens responded favourably to dietary restriction [72]. In a more recent study, 85% of offending food allergens in dogs with a delayed reaction to those allergens (> 6 h) were correctly identified by a lymphocyte proliferation test [73]. However, lymphocyte proliferation tests are currently not widely available commercially.

It is speculated that storage mites in dry dog foods might cause relapses of cAD because of allergenic cross‐reactivity with HDM, to which atopic dogs are frequently sensitised. However, there is currently no evidence that avoiding dry commercial dog foods benefits dogs sensitised to storage mites and/or HDM. Freezing dry dog foods might reduce contamination with storage mites, yet the impact of such freezing on clinical signs of mite‐sensitive dogs is unknown.

*Dermatophagoides* HDM glycoproteins are the most common allergens in dogs with cAD worldwide [74]. Reducing mite numbers and allergens in the home of mite‐hypersensitive patients is attractive in theory yet difficult in practice [75]. Controversy persists regarding the effectiveness of HDM control measures for human mite‐sensitive asthma or AD [76, 77, 78]. In households with atopic dogs, use of direct environmental flea control in the previous year alongside regular vacuuming was associated with a statistically significant reduction in mite allergen levels in living room carpets [79]. One uncontrolled study reported clinical improvement in mite‐sensitised atopic dogs following HDM control with a benzyl benzoate spray [80]. Additionally, isolation of dogs with cAD in cages where HDM were controlled led to rapid reductions in pruritus in most dogs with IgE hypersensitivity to environmental allergens [81].

Products (e.g., carpet sprays, powders, carpet shampoos, on‐animal products) containing ingredients other than benzyl benzoate are currently marketed in some countries and purport to reduce allergen levels in the household, or even on the pet. However, there is currently no evidence that these products provide any clinical benefit for dogs with cAD. Additionally, in a recent study, environmental Der f 1 concentration had an inverse association with TEWL, and no association with Canine Atopic Dermatitis Extent and Severity Index, 4th iteration (CADESI‐04) and Pruritus Visual Analogue Scale (PVAS) scores [82].

#### 6.2.3 Evidence of Safety

The various approaches to allergen avoidance are likely to be safe. However, clinicians should be aware of the potential for adverse effects from ectoparasiticides and other products used for controlling environmental allergens (which may include the humans and other animals in a household). Additionally, the potential for ecotoxicity (i.e., impact on nontarget organisms) should be considered when selecting ectoparasite and other products and treatment strategies.

#### 6.2.4 Recommendations

- In flea‐endemic regions, year‐round flea preventatives are recommended for all dogs with cAD as proactive therapy (SOR D). Standard‐of‐care flea control protocols should be followed according to regional guidelines. The efficacy of many flea preventatives may be reduced by frequent shampooing; careful selection and, if needed, oral or parenteral adulticides or more frequent application of topical adulticides are recommended in dogs that are washed repeatedly (SOR D).
- There is no evidence that serological or intradermal allergen tests for food allergens reliably predict the results of restriction–provocation dietary trials in dogs with adverse food reactions. Consequently, such tests cannot be recommended to assess food hypersensitivity in dogs with food‐associated cAD [68, 73] and elimination diets are recommended to evaluate the involvement of food allergens in cAD (SOR A).
- To decrease excessive storage mite contamination, owners should avoid storing dry foods in humid, warm areas and should use clean, sealed containers as proactive therapy (SOR D).
- If HDM avoidance measures are attempted, it would seem logical to restrict this to dogs sensitised to HDM alone, and to use a combination of measures that might include acaricides, impermeable pet mattress covers, and frequent and thorough pet mattress and environment washing and vacuuming.
- Any benefit is likely to take some months to occur owing to the long persistence of mite and other allergens in the environment. Environmental control of dust mite allergens therefore may only be suitable for proactive treatment in dust mite‐allergic dogs (SOR B).

### 6.3 Allergen (‐Specific) Immunotherapy

#### 6.3.1 Mechanism‐of‐Action

Allergen‐specific immunotherapy (AIT) involves the repeated administration of increasing doses of relevant allergens with the aim of reducing clinical signs of AD [83, 84]. AIT has a unique mechanism that may prevent disease progression. With time, it increases T‐regulatory cells and interleukin (IL)‐10 concentrations and may decrease allergen‐specific IgE [85].

#### 6.3.2 Evidence of Efficacy

AIT can be administered subcutaneously, percutaneously, intralymphatically or orally. Most studies have used subcutaneous protocols. Rush (or fast‐track) immunotherapy, in which the induction phase is shortened to a single day, follows the same mechanistic principles. More recently, sublingual, percutaneous and oromucosal approaches have been explored as alternative routes of allergen administration (Table S3).

Intradermal allergen tests (IDT) and IgE serology are useful to identify sensitisation to environmental allergens in dogs with cAD and to select relevant allergens for AIT [86, 87]. However, immediate IDT reactions and IgE serologies to environmental allergens are also common in atopic dogs, so these tests cannot differentiate cAD dogs from normal dogs [88, 89].

Veterinary surgeons may use either IDT or IgE serology to identify sensitisation to environmental allergens, as there is no clear evidence that AIT outcomes differ when allergens are selected by IDT versus serology [90, 91]. There is no standardisation in how IDT or IgE serology is performed to select allergens for AIT. Mounting evidence indicates that serological test results can vary substantially between laboratories [92]. To be included in AIT preparations, therefore, allergens should correspond to patterns of clinical exacerbation and likely exposure based on history and geographical location (taking allergen cross‐reactions into account).

Approximately 50%–80% of dogs receiving AIT for 6–12 months show good clinical improvement or a reduced need for antipruritic therapy [84, 93, 94]. In long‐term use, one third of owners administering AIT for 5–10 years rated it as very effective [95], and about 18% report complete remission without further therapy [96, 97]. In a long‐term retrospective survey, nearly two thirds of dogs exhibited satisfactory‐to‐excellent responses to AIT [98]. The QoL of atopic dogs and their owners was reported to improve on immunotherapy [99]. Owner compliance was identified as a major factor influencing the outcome of immunotherapy [100].

Rush immunotherapy has been reported to be as effective as conventional induction protocols [101, 102, 103, 104]. A pilot study of sublingual or oromucosal immunotherapy yielded promising results [105], yet a subsequent comparison of intralymphatic, subcutaneous and oromucosal AIT demonstrated markedly decreased efficacy of oromucosal immunotherapy compared with the other routes, although the case numbers were small [106]. By contrast, intralymphatic immunotherapy was shown in several studies to be at least as effective as, and potentially more effective than, subcutaneous AIT, particularly when performed under ultrasound guidance [104, 107, 108, 109].

Injection frequency and dose must be individualised based on clinical response and any adverse effects such as transient pruritus. If the patient's pruritus increases directly after the injection and gradually decreases again, the amount injected should be decreased. If the pruritus increases before the injection, it is advisable to shorten the time period between injections. Concurrently, the dose administered may be decreased.

Because AIT generally takes months to exert a clinical effect, concurrent anti‐inflammatory medication is usually required during the induction phase. Newer adjuvants and recombinant allergens may however reduce that time to clinical effect and improve the success rates [110]. There is no clear evidence in dogs that concurrent anti‐inflammatory therapy reduces AIT efficacy. AIT should usually be continued for ≥ 1 year before response is evaluated, and long‐term treatment is often needed [94]. Some dogs maintain remission after discontinuation, and in those with prolonged complete remission on AIT, attempts should be made to decrease the frequency of administration.

Readers are referred to detailed reviews for comprehensive discussion of AIT protocols and factors influencing treatment outcomes [84, 94].

#### 6.3.3 Evidence of Safety

Immunotherapy protocols have generally been reported as safe [94, 100, 108, 111]. Adverse effects are usually mild and transient, and include temporary increases in pruritus, although anaphylactic reactions very rarely can occur. The injection frequency and dose should be individualised according to clinical response and the occurrence of such adverse effects. There is no evidence to suggest that concurrent anti‐inflammatory or other medication reduces the safety of AIT.

#### 6.3.4 Recommendations

- A number of studies have evaluated AIT with very similar results. We can recommend this treatment for proactive therapy of cAD (SOR B).
- AIT should be recommended in dogs with AD when IDT or IgE serology has identified clinically relevant allergens, especially when avoidance is not feasible [112]. It also is indicated when symptomatic therapy is ineffective, poorly tolerated or not suitable for long‐term use [112] (SOR D).
- AIT will lead to complete remission in a minority of atopic dogs. However, based on available data, it will decrease pruritus, skin lesions and/or the need for additional medications in most dogs (SOR B).
- The length of time needed to see a clinical response means that AIT is only suitable for proactive maintenance treatment of cAD.

## 7 Anti‐Inflammatory, Antipruritic and Immunomodulating Therapy

### 7.1 Antihistamines

#### 7.1.1 Mechanism‐of‐Action

Type‐1 histamine receptor antagonists (H1‐antihistamines) include among others hydroxyzine, diphenhydramine, chlorpheniramine, dimetindene, clemastine, cetirizine and fexofenadine. These drugs act primarily by stabilising H1 receptors in an inactive state, thereby reducing histamine‐induced vasodilation, increased vascular permeability and sensory nerve stimulation.

#### 7.1.2 Evidence

A number of older studies did not have clear outcome measures and consequently were not evaluated. In one more contemporary study, approximately one quarter of owners perceived oral antihistamines as very effective in their atopic dogs [95]. However, placebo‐controlled trials with objective outcome assessments have shown limited efficacy (Table S4). Hydroxyzine failed to prevent lesion development in an experimental model of acute AD [113], and cetirizine did not significantly improve pruritus in dogs with cAD [114]. Clemastine, once considered effective, was subsequently shown to lack oral bioavailability [115].

Certain antihistamines demonstrate pharmacodynamic activity despite poor clinical efficacy. Cetirizine reduced histamine‐ and compound 48/80‐induced wheal formation in healthy dogs [116], and hydroxyzine and cetirizine inhibited intradermal histamine responses [117, 118]. Nevertheless, these physiological effects have not translated into consistent clinical improvements [116].

A randomised placebo‐controlled trial comparing dimetindene (Fenistil; Novartis) and a hydroxyzine/chlorpheniramine combination (Histacalmine; Virbac) demonstrated small but variable reductions in pruritus [119]. Fexofenadine produced a modest benefit, particularly when combined with vitamin E [120]. There is limited evidence that trimeprazine may reduce glucocorticoid requirements [121].

#### 7.1.3 Evidence of Safety

Antihistamines are generally safe with adverse effects limited to sedation and gastrointestinal upsets.

#### 7.1.4 Recommendations

- The desirable effects of antihistamines (mild antipruritic benefit and sedation) are frequently outweighed by limited efficacy and daily to twice‐daily dosing requirements.
- We cannot suggest antihistamines for reactive treatment of cAD (i.e., induction therapy to manage flares) (SOR B).
- They may be considered as proactive maintenance management of mild (SOR A) or seasonal disease, or when sedation is desirable (SOR D).
- Where possible, clinicians should use agents with documented pharmacological activity in dogs, specifically hydroxyzine (2 mg/kg per os twice daily) or cetirizine (0.5–1.0 mg/kg p.o. once daily) (SOR B).
- Clinicians should advise owners about the variable response, potential for sedation and lack of evidence for efficacy of topical antihistamines (SOR B).

### 7.2 Topical Glucocorticoids

#### 7.2.1 Mechanism‐of‐Action

Glucocorticoids affect cells by binding to the glucocorticoid receptor (GR). The activated GR‐glucocorticoid complex translocates to the nucleus (‘genomic effect’), where it activates transcription of anti‐inflammatory genes and at the same time represses transcription of pro‐inflammatory genes by interacting with transcription factors. Additionally, glucocorticoid actions are mediated through interactions with cytosolic GR or membrane‐bound GR, leading to modulation of the activity of various kinases and other inflammatory mediators (‘nongenomic effect’) [122]. This results in a rapid and broad‐spectrum reduction of acute inflammation with longer term treatment reversing chronic inflammatory changes.

#### 7.2.2 Evidence of Efficacy

Multiple RCTs and systematic reviews have confirmed the efficacy of topical glucocorticoids for treating cAD [4]. Two RCTs [123, 124] and a systematic review [4] provide evidence for the high efficacy of medium potency glucocorticoid sprays (i.e., 0.015% triamcinolone solution and 0.0584% hydrocortisone aceponate) for resolving skin lesions and pruritus in cAD (Table S5). In another 12‐week RCT, the hydrocortisone aceponate spray showed similar efficacy and tolerance compared to oral ciclosporin [125]. A small open study also showed that daily application of the hydrocortisone aceponate spray for 1–2 weeks significantly improved lesions and pruritus in atopic dogs [126] and the clinical responses correlated with decreased TEWL. In another small study, both daily and intermittent treatment on 3 days per week for 3 weeks improved CADESI‐03 lesion scores yet the effect was greater with daily treatment [81]. In an RCT of proactive therapy, treatment of atopic dogs with hydrocortisone aceponate spray on two consecutive days each week maintained remission and prevented relapses of pruritus and/or clinical lesions for a median of 115 days compared to 33 days for the placebo control [127]. Recently, a small, double‐blinded RCT showed that applying hydrocortisone aceponate (once daily for 7 days and then once every 2 days) in atopic dogs undergoing induction therapy with oclacitinib (i.e., 0.4–0.6 mg/kg twice daily for 14 days and then once daily for 14 days) significantly improved pruritus and lesion scores compared to the placebo [128].

Recent studies also have demonstrated efficacy of hydrocortisone aceponate in canine OE. In one RCT, hydrocortisone aceponate and prednisolone/polymyxin B/miconazole solutions administered daily were equally effective in resolving erythroceruminous otitis externa with staphylococcal and/or *Malassezia* overgrowth [129]. In an open study, following initial treatment with a betamethasone/ciprofloxacin/clotrimazole product, twice‐weekly proactive application of hydrocortisone aceponate maintained clinical remission in 88 of 90 treated ears over 12 months [130]. Similar results have been reported for other topical glucocorticoids. In a randomised, double‐blinded, vehicle‐controlled trial a 0.015% triamcinolone acetonide spray applied twice daily for 7 days, then once daily for 7 days and then every other day for 2 weeks (i.e., 28 days in total) significantly improved pruritus, skin lesions and overall assessment scores in atopic dogs [123]. In another RCT, twice‐weekly otic application of a 0.1% mometasone solution prevented flares and did not cause microbial dysbiosis in 10 atopic dogs [131].

In view of this evidence, it is likely that other topical glucocorticoid formulations will be beneficial in cAD, yet the efficacy and safety of these medications will vary with the type of glucocorticoid and vehicle used. It is also likely that appropriate topical glucocorticoid medications can be used to manage inflammation or pruritus at other sites in affected dogs (e.g., atopic conjunctivitis).

#### 7.2.3 Evidence of Safety

Clinicians should note that topical glucocorticoids are largely licensed for use over limited periods; although short‐term safety is very good, caution is advised with long‐term use as adverse effects are likely to occur. The risk with ‘traditional’ glucocorticoids (e.g., hydrocortisone, prednisolone, triamcinolone acetonide, betamethasone and dexamethasone) correlates with their anti‐inflammatory potency, which may be compounded by the vehicle (generally, more occlusive vehicles enhance potency and adverse effects). Local adverse effects include skin thinning with or without tearing, comedones and superficial follicular cysts (milia). Secondary staphylococcal and/or *Malassezia* infections also may occur and the resulting inflammation may mask the atrophic changes. Nevertheless, when carefully managed, an atrophic effect might be useful initially to reverse lichenification in chronically inflamed skin lesions. In addition to local effects, systemic absorption may result in hypothalamic–pituitary–adrenal (HPA) axis suppression and iatrogenic hypercortisolism. The risk is likely to vary with the glucocorticoid, vehicle and extent of application. For example, mild effects on adrenal function in healthy dogs were reported after twice‐weekly otic application of a dexamethasone and not of a betamethasone preparation [132]. One study reported that dexamethasone suppressed adrenocorticotropic hormone responses more than betamethasone, triamcinolone or mometasone, yet the differences were not significant [133]. An otic application of 0.1% dexamethasone for 2 weeks resulted in elevated liver enzyme levels and adrenal suppression in healthy dogs [134]; yet no effects were seen with 0.01% dexamethasone. Following 28 days of tapering application of a 0.015% triamcinolone acetonide spray, adverse effects were seen in five of 52 treated dogs (scaling/proliferation, polyphagia, polyuria and polydipsia, gastrointestinal upsets and minor reductions in total leucocyte, lymphocyte and eosinophil counts) [123].

Newer ‘nontraditional’ topical glucocorticoids (e.g., hydrocortisone aceponate, mometasone furoate and prednicarbate) have a better benefit/risk ratio with fewer local and systemic adverse effects compared to ‘traditional’ topical glucocorticoids of similar anti‐inflammatory potency. No adverse effects, cutaneous atrophy or significant HPA axis suppression were noted with hydrocortisone aceponate in safety studies and an RCT lasting up to 70 days [124, 135]. However, atrophic changes in the abdominal skin and suppression of IDT responses were seen in an experimental study [136]. In one 3‐week study, daily hydrocortisone aceponate treatment resulted in mild suppression of post‐ACTH cortisol levels yet no effect was seen with treatment for 3 days per week [137]. Additionally, no adverse effects applicable to treatment were noted in nine dogs treated concurrently with hydrocortisone aceponate and oclacitinib [128]. In other studies, otic application of mometasone furoate had no effect on IDT [138] and twice‐weekly otic treatment with a 0.1% mometasone furoate solution did not alter microbiome diversity or result in microbial overgrowth over 28 days in 10 atopic dogs [131]. Topical mometasone furoate, florfenicol and terbinafine in a depot formulation did not have an effect on ACTH stimulation testing in small breed dogs with bilateral OE [139]. However, absorption of topical glucocorticoids is related to the thickness of the epidermis and SC. Additionally, absorption of topical glucocorticoids is increased in inflamed skin [140].

#### 7.2.4 Evaluation of Safety

In general, short‐term safety is good. Long‐term safety is variably good to poor, depending on the benefit/risk ratio of the glucocorticoid, the extent of treated area, the thickness of the treated skin and the frequency of application. Although there are reports of contact reactions to topical glucocorticoid preparations, these may be to the vehicle and/or other ingredients rather than the glucocorticoid.

#### 7.2.5 Recommendations

- Topical glucocorticoids show rapid, lasting and broad‐spectrum anti‐inflammatory and antipruritic activity. They are therefore suitable for initial treatment of acute flares and chronic lesions as well as proactive maintenance therapy (SOR A).
- Topical glucocorticoids are ideally suited for localised skin lesions and/or inflammation at specific sites such as the ear canals and conjunctiva (SOR D).
- The anti‐inflammatory potency of nontraditional topical glucocorticoids may be limited in chronically lichenified skin (SOR D); ‘traditional’ topical glucocorticoids are more effective with these lesions.
- The type of topical glucocorticoid, treatment duration and frequency of use should be tailored to each patient (SOR D).
- Reactive therapy should normally continue until there is complete and stable remission of signs before switching to maintenance proactive therapy (SOR D).
- Where possible, nontraditional topical glucocorticoids should be used for long‐term treatment owing to their better safety profile (SOR D).

### 7.3 Systemic Glucocorticoids

#### 7.3.1 Mechanism‐of‐Action

Glucocorticoids bind to the glucocorticoid receptor, affecting transcription of target genes and modulating the activity of various kinases (see Section 7.2).

#### 7.3.2 Evidence of Efficacy

Systematic reviews of clinical trials in cAD [3, 4] and previous cAD guidelines [1, 2] have established the efficacy of oral glucocorticoids for reactive treatment of AD in dogs (Table S6). In most studies, oral prednisolone, prednisone or methylprednisolone given at 0.5–1.0 mg/kg per day, in one or divided into two doses, were used to induce clinical remission in atopic dogs [3, 4, 141]. Long‐acting injectable glucocorticoids have rarely been evaluated for efficacy in cAD.

Oral glucocorticoids improve pruritus and clinical signs (including acute and chronic lesions, otitis and pododermatitis) of dogs with severe or extensive cAD in the reactive phase of treatment. Because of the variability in the clinical presentation of cAD, study duration, glucocorticoid dosages and tapering regimens included in these studies, a meta‐analysis of pooled glucocorticoid study data was not possible. Most glucocorticoid studies in cAD utilised a predetermined dose‐tapering schedule of daily administration for 7 days followed by tapering to every other day for 14–21 days [3, 4, 141]. Unfortunately, there have been no controlled comparative clinical trials evaluating the efficacy of different glucocorticoid dosages and dosage‐tapering schemes for the control of clinical signs in dogs with cAD.

Oral glucocorticoids (prednisolone, prednisone or methylprednisolone) should be used at 0.5 mg/kg once to twice daily until inducing remission (reactive phase) of clinical signs of cAD, including if needed, during an elimination diet trial for diagnosis of food‐associated cAD [1, 142]. After clinical remission occurs, the dose of oral glucocorticoids should be tapered (lower dosages and frequency) to minimise the risk of adverse effects and then discontinued. To avoid long‐term adverse effects of oral glucocorticoids, other interventions with fewer adverse effects and previously established clinical efficacy in cAD (e.g., proactive topical glucocorticoid therapy, lokivetmab, Janus kinase [JAK] inhibitors, ciclosporin and others) can be used to manage ongoing or flares of pruritus or inflammation while eliminating or addressing the AD‐associated flare factors in the individual patients.

Oral glucocorticoids can be concomitantly given with oral ciclosporin, oclacitinib or lokivetmab during the reactive phase of cAD therapy; however, clinicians should consider the benefit of utilising such anti‐inflammatory/antipruritic combinations when glucocorticoids are deemed to have the most extensive ‘breadth of immune inhibition’ in cAD [7]. In the patients where ciclosporin therapy will be used initially to induce clinical remission of cAD, combined oral prednisolone for the first 3 weeks increases the speed of onset of clinical improvement compared to ciclosporin alone [143]. A course of 0.5 mg/kg prednisolone twice daily for the first 4 days of oclacitinib treatment also may reduce the rebound of pruritus that can be seen when switching from administration every 12 h to every 24 h (see Section 7.4.1) [144].

No data were available to assess the efficacy of monotherapy with systemic glucocorticoids in dogs during the proactive therapy of cAD.

#### 7.3.3 Evidence of Safety

Adverse effects of oral glucocorticoids (e.g., polyuria, polydipsia, polyphagia, panting) are common in dogs. Although short‐term administration of oral glucocorticoids (e.g., prednisone) over 3 weeks in systemically otherwise healthy atopic dogs showed no significant changes in blood glucose concentration, serum sodium or potassium concentration, or echocardiographic indices of wall thickness or cardiac function [145], glucocorticoids should be carefully used in cAD patients with congestive heart failure.

Long‐term use of glucocorticoids can result in iatrogenic hypercorticolism, the onset of which is drug‐, dose‐, frequency‐, duration‐ and patient‐dependent. The clinical signs may include erythematous and pruritic skin lesions associated with calcinosis cutis. Owners may interpret this as a flare of their dog's cAD, thereby prompting them to inappropriately increase the dosage and/or frequency of use of glucocorticoids. Long‐term usage of glucocorticoids also has been associated with predisposition to subclinical bacteriuria and urinary tract infections [146, 147]; however, a recent prospective study found no predisposition to this in dogs that received antineoplastic chemotherapy which included prednisone [148]. Nevertheless, signs of iatrogenic hypercortisolism (especially calcinosis cutis) should prompt clinicians to taper the glucocorticoids as quickly as possible and institute alternative measures to manage the cAD.

Long‐acting injectable glucocorticoids should be avoided wherever possible as the lack of ability to taper their dose increases the risk of adverse events.

#### 7.3.4 Recommendations

- Oral glucocorticoids are recommended for the reactive treatment of flares owing to their rapid action and high efficacy (SOR A) yet should be used with caution as long‐term treatment owing to the common occurrence of adverse effects.
- Atopic dogs without a rapid clinical benefit during oral glucocorticoid administration should be re‐evaluated for presence of secondary infections and complications (e.g., ectoparasitism) and/or alternative diagnoses (e.g., cutaneous epitheliotrophic T‐cell lymphoma) (SOR D).
- Oral glucocorticoids can be concomitantly given with oral ciclosporin, oclacitinib or lokivetmab during the reactive phase of cAD therapy (SOR B).
- Prolonged concomitant administration of oral glucocorticoids, ciclosporin or oclacitinib in any combination is not recommended because of the theoretical higher risk of immunosuppression predisposing to potentially severe opportunistic infections of the skin or other organs, as well as the development of neoplasia (SOR D).

### 7.4 JAK Inhibitors

#### 7.4.1 Oclacitinib

##### 7.4.1.1 Mechanism‐of‐Action

Oclacitinib (Apoquel; Zoetis) is a receptor blocker that inhibits JAK1 twice as much as JAK2 and consequently predominantly blocks the activity of IL‐31 and other cytokines that use the JAK‐Signal Transducers and Activators of Transcription (STAT) pathway (e.g., T‐helper (TH)2 and TH22 cytokines).

##### 7.4.1.2 Evidence of Efficacy

Multiple experimental studies and RCTs have shown that oclacitinib rapidly reduces pruritus and clinical signs of cAD significantly better than placebo [149, 150] and at least as well as prednisolone [141] and ciclosporin [151] (Table S7). Oclacitinib is licensed for the treatment of pruritus associated with allergic dermatitis and the clinical manifestations of cAD in dogs at 0.4–0.6 mg/kg every 12 h for 14 days and then every 24 h. A flare of pruritus may be seen after transition to dosing every 24 h. This effect may be reduced when oclacitinib is associated with prednisolone at 0.5 mg/kg p.o. twice daily for the first 4 days of administration [144]. It is possible that the administration of oclacitinib every 12 h for longer than 14 days may ameliorate this flare phenomenon and achieve remission in dogs that cannot be maintained on the ‘every 24 h’ regimen [152]. Once complete remission is achieved, further tapering of the dose and/or frequency of treatment needed to maintain the remission of signs may be possible. In an RCT with 18 atopic dogs, concurrent topical treatment with a 0.0584% hydrocortisone aceponate spray helped prevent flares of pruritus and inflammation when tapering oclacitinib therapy in dogs with cAD [128].

##### 7.4.1.3 Evidence of Safety

Short‐term adverse effects of oclacitinib appear minor [141, 149, 150, 151]. Long‐term administration of oclacitinib at the licensed dose is well tolerated [153]. Changes in haematological and biochemical panels, and urinalysis parameters seem minimal even after prolonged treatment at either every 24 h (≤ 630 days) [153] or every 12 h (≤ 113 days) dosing [152]. Although there is no consensus on the frequency, periodic monitoring of serum biochemical results and, particularly, complete blood counts of dogs on long‐term treatment is recommended, especially with higher‐than‐licensed doses.

Oclacitinib is not approved for use in dogs < 12 months of age or with concurrent neoplastic conditions (including histiocytoma and papillomas). It is possible that treatment may increase susceptibility to infections, although treatment has not specifically been associated with urinary tract infections [153] and resulted in less systemic antimicrobial use for skin infections in one small study [154]. However, otitis, *Malassezia* dermatitis, pyoderma and interdigital furunculosis are among the listed possible adverse effects (although those may be associated with a flare of the underlying atopic or allergic condition and not be a direct adverse effect of the drug). Behavioural changes (e.g., lethargy) have been reported in dogs on oclacitinib. Although anecdotal, a possible increased aggressive behaviour, weight changes and reduced response to treatment of neoplastic diseases in dogs receiving oclacitinib have been reported.

##### 7.4.1.4 Recommendations

- Oclacitinib shows rapid and lasting antipruritic and anti‐inflammatory activity and is therefore suitable for treatment of acute flares and proactive maintenance therapy of cAD (SOR A).
- The activity of oclacitinib is likely to be semi‐broad spectrum and, therefore, dogs with chronic lesions (particularly otitis and pododermatitis) likely to involve non‐JAK/STAT‐associated mediators may benefit from additional or alternative therapies to induce and maintain clinical remission (SOR D).
- Regular clinical monitoring with haematological, biochemical and urinalysis results is recommended especially in dogs maintained on every 12 h treatment, or with underlying conditions and/or concurrent treatments that make them more susceptible to immunosuppression or other adverse events (SOR C).

#### 7.4.2 Ilunocitinib

##### 7.4.2.1 Mechanism‐of‐Action

Ilunocitinib (Zenrelia; Elanco) is a pan JAK receptor blocker that inhibits the activity of inflammatory cytokines that use the JAK–STAT pathway. Because of the more extensive JAK receptor inhibition, ilunocitinib may have a broader activity against cAD than other commercially available JAK inhibitors.

##### 7.4.2.2 Evidence of Efficacy

Three RCTs have been published on the effect of ilunocitinib in the treatment of cAD [155, 156] or allergic dermatitis [157] (Table S7). Two of these studies were placebo‐controlled [156, 157] and one was a head‐to‐head study comparing ilunocitinib to oclacitinib [155]. In the placebo‐controlled clinical trials, ilunocitinib, administered at the label dose of 0.6–0.8 mg/kg once daily, showed a significant improvement in pruritus and clinical signs of cAD and allergies as early as D2 postadministration, and reduction in pruritus or clinical signs of ≥ 50% was seen in 25.4% and 83% of the dogs after D7 [155] and D28 [156], respectively. In the head‐to‐head double‐blinded RCT, ilunocitinib, administered at the above dose, lowered both the pruritus and clinical scores significantly more than oclacitinib (both drugs were administered at the labelled doses) after D14 and D28, respectively, and this effect persisted until the end of the study (D112) [155]. Because of the single daily dosing regimen, the deterioration seen occasionally with oclacitinib when its dose is reduced to once daily (see Section 7.4.1) is not seen with ilunocitinib administration.

##### 7.4.2.3 Evidence of Safety

Short‐term adverse effects of ilunocitinib appear minor [155, 156, 157] even when given at 2–3 times the label dose for 6 months [158]. In a safety study where ilunocitinib was administered up to five times the label dose for 6 months, changes in haematological and biochemical panels, and urinalysis parameters were minimal‐to‐absent [158]. Similar results were confirmed in the field studies [155, 156, 157]. Periodic monitoring of complete blood counts and serum biochemical results of dogs on long‐term treatment is recommended, although there is no consensus on the frequency.

Unfortunately, because of the recent introduction of ilunocitinib to the market, long‐term (> 6 months) safety data are not available.

As for oclacitinib, ilunocitinib is not approved for use in dogs < 12 months old or with a serious infection. It is possible that treatment may increase susceptibility to infections, although treatment has not specifically been associated with any infections [155, 156, 157]. The most commonly reported adverse effects include dermatitis (although this may be associated with a flare of the underlying atopic or allergic condition and not be a direct adverse effect of the drug) and gastrointestinal disorders. Immunological disorders were reported in < 1% of the dogs enrolled in clinical studies [155, 156]. Similar adverse effects between ilunocitinib and oclacitinib were seen in the head‐to‐head clinical trial [155].

In the USA, the FDA recommends discontinuing ilunocitinib for ≥ 28 days (and ≤ 3 months) before vaccination and for another ≤ 28 days after vaccination to allow an adequate immune response based on a laboratory study in unvaccinated puppies [159]. However, a recent study administering ilunocitinib, at label and a threefold higher dose, to 10–11‐month‐old already vaccinated Beagles showed no difference in antibody titres for the major vaccines, including rabies, 15 and 28 days after booster administration [160]. The recommendation to cease treatment around vaccination is not required in the European Union, United Kingdom and other countries. It is the responsibility of clinicians to check the regulatory requirements in their country of practice.

##### 7.4.2.4 Recommendations

- Ilunocitinib shows rapid and lasting anti‐itch and anti‐inflammatory activity, and is therefore suitable for treatment of acute flares and proactive maintenance therapy of cAD (SOR A).
- The activity of ilunocitinib is possibly broader than other commercially available JAK/STAT inhibitors (i.e., oclacitinib and atinvicitinib) and therefore may be more effective in the treatment of cAD (SOR B) potentially even in dogs not responding to other JAK inhibitors (SOR D).
- Regular clinical monitoring with haematological, biochemical and urinalysis results is recommended, particularly with underlying conditions and/or concurrent treatments that render dogs more susceptible to immunosuppression or other adverse events (SOR D).
- The response to treatment in dogs with chronic lesions (particularly otitis and pododermatitis) should be monitored carefully. Lesions likely to involve non‐JAK/STAT‐associated mediators may need additional or alternative therapies to induce and maintain clinical remission (SOR D).

#### 7.4.3 Atinvicitinib

##### 7.4.3.1 Mechanism‐of‐Action

Atinvicitinib (Numelvi; MSD/Merck) is a second‐generation JAK1‐selective receptor blocker that inhibits the activity of cytokines, such as IL‐4, IL‐13 and IL‐31, that use the JAK1‐STAT pathway and consequently the treatment of pruritus associated with allergic dermatitis in dogs.

##### 7.4.3.2 Evidence of Efficacy

Atinvicitinib is licensed for the treatment of pruritus associated with allergic dermatitis and the clinical manifestations associated with cAD in dogs at a dose of 0.8–1.2 mg/kg once daily. However, other than four abstracts [161, 162, 163, 164], there are no published studies on the clinical efficacy of atinvicitinib in the treatment of cAD (Table S7). In both abstracts, atinvicitinib was administered at a dose of 0.8–1.2 mg/kg once daily for 28 days. Compared to placebo, a significant reduction in pruritus was seen in the treatment group on D1 to D7 [164]. After 28 days of administration, atinvicitinib showed a reduction of ≥ 50% of pruritus or clinical scores in 87.5% of dogs when given at 0.8–1.2 mg/kg once daily, and in 73.3% of dogs given 0.4–0.6 mg/kg twice daily for 14 days and then every 24 h for another 14 days, compared to 23.1% of dogs in the placebo group [164]. In a more recent abstract, atinvicitinib significantly reduced IL‐31‐induced pruritus 2–4 h after dosing and was at least as effective as oclacitinib and ilunocitinib [161]. In a further abstract, 302 allergic dogs were treated with atinvicitinib for 30 days and resolution of clinical signs was reported in 78% of the 214 dogs re‐examined after 30 days [162].

##### 7.4.3.3 Evidence of Safety

Short‐term (28‐day) adverse effects of atinvicitinib appeared minor in two placebo‐controlled clinical trials, only published in abstract form [163, 164]. The most reported adverse effects included gastrointestinal abnormalities and lethargy. There are no restrictions around vaccination or a recommendation for periodic blood tests. In one study abstract, 40 Beagles 6 months of age were administered atinvicitinib at one‐, three‐ and fivefold the registered dose or placebo daily for 6 months [165]. No effects of treatment were noted on food consumption, body weight, haematological, clinical chemical, and urinalysis results, faecal evaluations, bone marrow smears or physical examinations, except in one dog administered 6.0 mg/kg (fivefold normal) once daily with clinical signs starting after 18 weeks.

However, it is not licensed for use in dogs < 6 months of age and caution is recommended in dogs on treatment with other potentially immunosuppressive agents (including glucocorticoids and ciclosporin) or with immunosuppressive conditions.

As for ilunocitinib, unfortunately, because of the recent introduction of atinvicitinib to the market, long‐term (> 6 months) safety data are not available. In addition, at this time to the best of the authors' knowledge, there are no peer‐reviewed publication data available for atinvicitinib.

##### 7.4.3.4 Recommendations

- Atinvicitinib seems to have a rapid and lasting anti‐itch activity and seems suitable for treatment of acute flares and proactive maintenance therapy of cAD (SOR C).
- Dogs with chronic lesions (particularly otitis and pododermatitis) likely to involve non‐JAK/STAT‐associated mediators may benefit from additional or alternative therapies to induce and maintain clinical remission (SOR D).
- Regular clinical monitoring with haematological, biochemical and urinalysis findings is recommended in dogs with underlying conditions and/or concurrent treatments that make them more susceptible to immunosuppression or other adverse events (SOR D).
- Owing to a lack of published data, no further recommendations are possible.

### 7.5 Lokivetmab

#### 7.5.1 Mechanism‐of‐Action

Lokivetmab (Cytopoint; Zoetis) is a caninised anti‐canine IL‐31 monoclonal antibody used at 1 mg/kg subcutaneously every 4 weeks (Europe) or 2 mg/kg s.c. every 4–8 weeks (USA) [166]. It specifically binds to canine IL‐31 and blocks its cell‐signalling. As IL‐31 is a cytokine that plays an important role in the pathogenesis of cAD, lokivetmab was the first biological therapy to be approved in veterinary medicine for the treatment of cAD.

#### 7.5.2 Evidence of Efficacy

Various studies have shown that lokivetmab rapidly reduces pruritus, and to a lesser extent skin lesions in cAD (Table S8) [167, 168, 169, 170, 171, 172, 173, 174, 175]. Lokivetmab also improves the QoL of pet owners [173]. It can be administered regularly at the above dosages until complete remission of signs is obtained.

Lokivetmab reduced pruritus and clinical signs significantly better than placebo in allergic dermatitis sensu lato [176, 177], cAD sensu stricto [174, 178] and in a canine model of IL‐31‐induced pruritus [168]. It seems more antipruritic than anti‐inflammatory by decreasing serum IL‐31 concentrations [167]. The antipruritic activity starts within hours of treatment and lasts for ≤ 56 days [166, 178], and it was more quickly effective for decreasing pruritus (not skin lesions) than ciclosporin in one study [178]. The degree and duration of the response increase with the dose [166, 168], although in a recent study even at the highest dosage of 2 mg/kg, the duration of activity was only 42 days [168]. The maximum effect seems to be obtained after the second injection [175]. In a 9‐month study, pruritus decreased further with regular injections and reached a plateau after four consecutive monthly injections [178]. In another 12‐month study, it was observed that dogs with severe/very severe pruritus before initiation of therapy and large/giant‐sized dogs had higher odds of treatment success [176]. Furthermore, dogs that previously did not respond to oclacitinib were less likely to respond to lokivetmab injections [176]. One study showed that the probability of treatment failure decreased with increasing treatment duration. In the same study, the treatment outcome was not influenced by age, disease chronicity, dosage and/or secondary infection [171].

The co‐administration of topical treatments designed to restore the defective skin barrier may help to improve the antipruritic and anti‐inflammatory effects of lokivetmab and extend the time‐to‐flare [28]. Furthermore, regular injections of lokivetmab may reduce the use of other products for treating atopic dogs in ≤ 80% of cases and facilitate the care for owners [170].

In an AD model, one single injection of lokivetmab prevented pruritic manifestations yet interestingly not clinical lesions of dogs experimentally sensitised to HDM [179]. In the same study, the time‐to‐flare was delayed by 63 days and one quarter of the treated dogs did not exhibit a flare for ≥ 1 year [179]. Lokivetmab also has been tested as an alternative proactive therapy to maintain clinical remission. Clinical flares were largely prevented when lokivetmab was given 1 h before allergen challenge in a colony of atopic Beagle dogs [172].

#### 7.5.3 Evidence for Safety

Both short‐term and long‐term adverse effects of lokivetmab seem minor in healthy [180] and atopic or allergic dogs [166, 176, 177, 178]. Reported adverse effects include vomiting, diarrhoea, allergic reactions and seizures. Owing to its unique MoA, lokivetmab can theoretically be used safely in dogs treated with a wide variety of other medicines and vaccines [176, 177, 178]. Treatment‐induced immunogenicity (increase in anti‐lokivetmab antibody titres > 10‐fold compared to pre‐dose titres) occurs in 2%–2.5% of cases [166, 178]. This may explain why certain dogs cease responding to lokivetmab after their second or subsequent doses [176], yet anaphylactic or immune‐complex‐associated reactions have not been reported. Adverse effects are seen in < 10% of dogs; the major adverse effect is lethargy [171, 176].

#### 7.5.4 Recommendations

- Lokivetmab shows rapid and lasting antipruritic and, to a more variable extent, anti‐inflammatory activity and is therefore suitable for treatment of acute flares and proactive maintenance therapy, especially in dogs where pruritus is the main clinical problem (SOR A).
- The activity of lokivetmab is likely to be narrow spectrum and therefore dogs with chronic lesions, otitis and/or pododermatitis will benefit from additional therapy to induce clinical remission (SOR D).
- Short‐ and long‐term safety is good [171, 176]; lokivetmab is therefore suitable for combination therapy with other anti‐inflammatory agents and/or where concurrent conditions and treatments preclude the use of other anti‐inflammatory agents (SOR C).

### 7.6 Ciclosporin

#### 7.6.1 Mechanism‐of‐Action

Ciclosporin is an oral calcineurin inhibitor which forms a complex with cyclophilin to block the phosphatase activity of calcineurin, which in turn decreases the production of inflammatory cytokines by T‐lymphocytes.

#### 7.6.2 Evidence of Efficacy

Ciclosporin has been used for over four decades in the treatment of allergic diseases in dogs and cats with significant success in ameliorating the clinical signs and improving the QoL of pets and their owners (Table S9). In veterinary medicine, the first approved ciclosporin (modified) microemulsion formulation (Atopica; Elanco Animal Health) is labelled at a starting dosage of 5 mg/kg once daily and continued at this dosage until a halving or a satisfactory decrease of severity of signs is achieved. The onset of clinical benefit normally requires 2–4 weeks of treatment (and up to 6 weeks in some cases) [178, 181]. After this improvement is reached, the dose should be decreased by either increasing dosage intervals (e.g., going from every day to every other day) or by decreasing the daily dose by half. After a further reduction of signs of approximately 75%, the administration could be further reduced to twice weekly or a 75% reduction of the original daily dose. Further dose titration can continue to the minimal effective dose and/or frequency, although this is rarely achievable in practice. Generic veterinary formulations of modified ciclosporin, shown to be bioequivalent to Atopica (Elanco Animal Health), are acceptable substitutes for it and are currently available in both Europe and North America (Cyclavance [Virbac]; Modulis (Ceva); Sporimune [Dechra]). Because of the slow onset of clinical efficacy, dose or frequency adjustments should not be made for 1 month after starting the treatment protocol. To alleviate the clinical signs of cAD during ciclosporin's lag phase, the concurrent use of glucocorticoids (prednisolone at 1 mg/kg once daily for 7 days followed by alternate day dosing for 14 days) [143] or oclacitinib (0.4–0.6 mg/kg twice daily for 14 days, then once daily for 7 days) [182] during the first 2–3 weeks of ciclosporin administration may be beneficial. The combination with prednisolone led to a quicker improvement of the clinical signs than when ciclosporin was given alone. Additional adverse effects over those expected for prednisolone, oclacitinib and ciclosporin were not reported. A human generic formulation of ciclosporin formulation (Equoral; Teva) given at 5 mg/kg once daily for 42 days was shown to be as effective as prednisone (1 mg/kg once daily for 7 days followed by alternate day dosing for 35 days) in reducing clinical signs of cAD in dogs [183]. The same human generic formulation was recently shown to have similar bioequivalence (measured as whole blood ciclosporin concentrations) to Atopica after a single oral administration in a randomised, blinded, cross‐over study, after a single administration in eight healthy research beagle dogs [184]. Please note that it is the responsibility of clinicians to check the regulatory requirements governing the use of human generic formulations where there are licensed veterinary formulations in their country of practice.

#### 7.6.3 Evidence for Safety

Minor adverse events (e.g., vomiting and diarrhoea) are common after initiating ciclosporin therapy; most improve spontaneously upon further administration of this drug [181, 185]. Such adverse events can be easily reversed and/or ameliorated by freezing the capsule formulation (for vomiting) [185, 186], administering the drug with food (vomiting and diarrhoea) [187] or increasing the fibre content in the diet (diarrhoea). Additional adverse effects over those expected for prednisolone, oclacitinib and ciclosporin were not reported in studies of concomitant administration for ≤ 3 weeks (see above). However, concurrent long‐term administration of oral ciclosporin, glucocorticoids and/or JAK inhibitors (especially at higher dosages of either or both drugs) should be monitored carefully, as combined immune suppression is likely to result in a higher risk for development of potentially severe opportunistic infections of the skin or other organs. Ciclosporin is not recommended in dogs with a history of malignant tumours.

Ciclosporin is metabolised in the liver by the hepatic cytochrome P‐450 3A enzymes, and interactions with drugs likewise metabolised are reported. Examples are doxycycline, erythromycin, fluconazole, ivermectin, ketoconazole, phenobarbital, rifampicin and terbinafine [188].

There is no consensus on the need for laboratory monitoring (e.g., haematological, serum biochemical and urinalysis results) or drug concentration monitoring during prolonged ciclosporin administration. However, such tests should be performed if signs of systemic illness develop or in dogs at greater risk of adverse effects or infections or when an absorption problem is suspected owing to a lack of clinical response.

#### 7.6.4 Recommendations

- Ciclosporin shows a delayed although long‐lasting anti‐inflammatory and antipruritic activity; thus, it is more suitable for proactive maintenance therapy of cAD and not recommended for reactive therapy (SOR A).
- It shows broad‐spectrum anti‐inflammatory activity and therefore should be effective for acute and chronic skin lesions and pododermatitis, although the efficacy in otitis is less clear (SOR D).
- Canine‐specific generic formulations and a specific human generic formulation have shown bioequivalence to the brand‐name ciclosporin (SOR B). For this reason, similar clinical results are expected (SOR D).
- Regular monitoring with haematological, biochemical and urinalysis findings is controversial and potentially unnecessary in the absence of systemic clinical signs or patient‐associated risk factors (SOR D).
- Although the concomitant administration of other immunomodulatory treatments is recommended in the short term (SOR B), the need for long‐term combination therapy needs to be balanced against the risk of adverse effects, especially immunosuppression (SOR D).

### 7.7 Tacrolimus

#### 7.7.1 Mechanism‐of‐Action

Tacrolimus is a broad‐spectrum anti‐inflammatory topical calcineurin inhibitor with similar properties to ciclosporin.

#### 7.7.2 Evidence of Efficacy

In atopic dogs, 0.1% tacrolimus ointment has been shown to be effective, especially in dogs with localised disease [189, 190]. The efficacy appears highest when used twice daily for 1 week (or to remission) and then reduced to the frequency needed to control the clinical signs. However, as for ciclosporin, the relatively slow improvement seen with tacrolimus suggests that it is not suitable for treating acute flares of cAD.

#### 7.7.3 Evidence of Safety

As in humans with AD, application of tacrolimus might be associated with signs suggestive of mild irritation [189]. However, other adverse effects have not been reported.

#### 7.7.4 Recommendations

- Topical 0.1% tacrolimus shows lasting broad‐spectrum anti‐inflammatory and antipruritic activity, yet the onset of efficacy is slow. It is therefore suitable for proactive maintenance therapy and not for reactive treatment of flares (SOR B).
- It shows broad‐spectrum anti‐inflammatory activity and therefore should be effective for acute and chronic lesions (SOR D).
- It is ideally suited to localised skin lesions and/or inflammation at specific sites such as the interdigital skin and conjunctiva (SOR D).
- Short‐ and long‐term safety is good; 0.1% tacrolimus is therefore suitable for combination therapy with other anti‐inflammatory agents and/or where concurrent conditions and treatments preclude the use of other anti‐inflammatory agents (SOR D).
- The treatment duration and frequency of use should be tailored to each patient; applications should normally continue twice daily until there is complete and stable remission of signs before switching to maintenance therapy (SOR D).

### 7.8 Other Immunomodulators

Two RCTs provide evidence of the efficacy of recombinant canine gamma‐interferon to treat dogs with cAD in Japan [191, 192]. Suggested effective dosages are 5000 to 10,000 units subcutaneously three times weekly for 4 weeks and then once weekly. Adverse effects appear to be minimal [191, 192]. Two small uncontrolled studies suggest that subcutaneous injections of recombinant feline omega interferon (Virbagen Omega; Virbac) might have clinical efficacy to treat dogs with cAD [193, 194]. Suggested doses of 1–5 million units three times weekly for 4 weeks and then every month appear to be well tolerated. Another RCT showed some inconsistent and mild improvement in skin lesions and pruritus after either subcutaneous injections or oral administration of feline interferon‐omega [195]. Whether or not repeated injections of recombinant feline omega interferon in dogs lead to a host immune response against this heterologous protein, later followed by a progressive reduction in this biological drug's efficacy is unknown. One small uncontrolled study demonstrated a clinical benefit of oral use of 30 mg/kg once daily of a modified‐release formulation of mycophenolate in atopic dogs over an 84‐day trial. In that study, a tapering dose of prednisolone was administered concomitantly for the first 28 days [196]. Further recommendations for these interventions must await the publication of larger and longer clinical trials.

### 7.9 Essential Fatty Acids and Other Nutritional Supplements

EFA supplements, EFA‐enriched diets and other nutritional supplements are considered in Section 4.3. under Skin Barrier Care.

### 7.10 Other Interventions

A large RCT confirmed that masitinib (Masivet/Kinavet; AB Science) at 12.5 mg/kg once daily was moderately effective in reducing clinical signs in atopic dogs. The development of a protein‐losing nephropathy in some dogs—which, if unrecognised could be potentially fatal—is a limitation of masitinib treatment [197]. Masitinib might be an alternative for atopic dogs with signs not responding to other approved drugs (SOR C).

An open RCT evaluating pentoxifylline at the dose of 20 mg/kg every 8 h, either alone or in combination with oral EFA supplementation, reported a significantly greater improvement in skin lesions and pruritus of these interventions over placebo; the effect seemed highest for dogs treated with the combination of pentoxifylline and EFAs (SOR C) [198].

A small proof‐of‐concept trial reported the clinical benefit and relative safety of low‐dose once‐weekly oral methotrexate for treatment of cAD [199].

Treatment of allergic pododermatitis in dogs with a novel transient receptor potential vanilloid 1 (TRPV1) channel antagonist improved clinical signs and QoL, yet the study did not have a control group [200].

An RCT showed no benefit of low‐level laser therapy in dogs with localised pedal AD (SOR B) [201]. Likewise, the selective serotonin reuptake inhibitor (SSRI) fluoxetine, given at 1 mg/kg p.o. once daily, showed no clinical efficacy in a small RCT of dogs with cAD (SOR B) [202]. Other drugs appear to provide little (misoprostol, tepoxalin) or no benefit (e.g., leukotriene inhibitors, capsaicin, dextromethorphan) [4].

## 8 Summary

Since the original ICADA treatment guidelines for cAD in 2010 and the minor revision in 2015, our understanding of the disease and its treatment has undergone a profound shift. Fundamental to this has been a move from regarding AD as a single disease entity with an allergic aetiology to a view that reflects the genetic, aetiological, immunological and clinical complexity of the condition. We now recognise that affected dogs will benefit from a longer term integrated approach that involves initial Phase 1 treatment of existing inflammation and pruritus followed by regular and long‐term Phase 2 proactive therapy to maintain the clinical remission and prevent flares. In humans with AD, there is evidence of high benefit, cost‐effectiveness and low risk of proactive intermittent applications of topical glucocorticoids and tacrolimus to skin areas repeatedly affected during flares of AD [203, 204, 205, 206]. Such intermittent applications of potent anti‐inflammatory drugs onto healed skin appear to delay or prevent flares of AD skin lesions. A small RCT tested the efficacy of a hydrocortisone aceponate spray applied to previously affected areas on two consecutive days after lesions had been controlled with the same spray. The time to recurrence of flares at these sites was nearly fourfold longer (median 115 days) in dogs intermittently treated with the topical glucocorticoids compared to those sprayed with placebo (SOR B) [127]. In a similar study, lokivetmab was used as long‐term monotherapy in dogs with spontaneous cAD previously controlled with other medications. The average time to a flare was 63 days and a fourth quarter of the dogs did not experience any flare within the 1‐year follow‐up [179]. It is therefore likely that proactive therapy will benefit atopic dogs. In particular, preventing flares of pruritus and inflammation is likely to prevent the development of chronic changes that are harder to manage and/or may require surgical intervention (SOR D).

Phase 1 reactive treatment generally requires rapid‐acting and broad‐spectrum drugs such as glucocorticoids, yet rapid‐acting semi broad‐spectrum (e.g., JAK inhibitors) or narrow‐spectrum drugs (e.g., lokivetmab) could be used in pruritic dogs with only acute or mild‐to‐no skin lesions. It is important that therapy is continued at an effective dose until the skin lesions and pruritus have completely resolved before adjusting treatment, even at the cost of some short‐term adverse effects. This reduces the risk of a rapid rebound flare once treatment is tapered.

Phase 2 proactive treatment can be considered once the clinical signs are fully in remission. A rapid onset of action is not required, which allows a greater range of treatment options to be considered. These should be aimed at the suspected triggers in each dog. Options include skin barrier care, microbiome modulation, dietary or environmental allergen avoidance, AIT, ciclosporin and tacrolimus, although additional anti‐inflammatory and antipruritic therapy may be required. Proactive therapy with topical glucocorticoids can be used to manage ongoing subclinical inflammation in the skin, paws (for chronic interdigital furunculosis) and ear (for OE) or eyes (for conjunctivitis). Narrow‐ or single‐target drugs (e.g., lokivetmab) may be most effective in this phase in the absence of more complex networks of inflammation. Regular treatment may block key itch and inflammation pathways thereby preventing flares. Where is this is insufficient, long‐term treatment with broader‐spectrum agents such as JAK‐inhibitors or ciclosporin can be considered.

It is hoped that these guidelines will provide veterinary surgeons with the information that they need to select the most appropriate treatment(s) for their patients, taking into account the stage of their disease, its severity and the distribution of their lesions. Veterinary surgeons should remember to discuss the likely benefit, potential adverse effects, ease of administration and costs of each recommended intervention alone or in combination. Ultimately, the QoL of both dogs and their owners (including their preferences and concerns) should be considered before a treatment plan is designed and implemented.

## Author Contributions

**Ralf S. Mueller:** conceptualization, investigation, writing – original draft, validation, writing – review and editing, project administration, methodology. **Frane Banovic:** conceptualization, investigation, writing – original draft, methodology, validation, writing – review and editing. **Emmanuel Bensignor:** conceptualization, investigation, writing – original draft, writing – review and editing, validation, methodology. **Domenico Santoro:** conceptualization, investigation, writing – original draft, methodology, validation, writing – review and editing. **Tim Nuttall:** conceptualization, investigation, writing – original draft, methodology, validation, writing – review and editing, project administration.

## Funding

This work was self‐funded; ICADA annual meetings are supported by the World Association for Veterinary Dermatology.

## Conflicts of Interest

R.M. acted as a consultant or received support for studies or lectures from Artuvet, Boehringer Ingelheim Animal Health, Ceva Animal Health, Elanco, Greer Laboratories, Heska Laboratories, Hill's, Royal Canin, MSD Animal Health, Nextmune, Synlab, Virbac Animal Health and Zoetis. F.B. has been an investigator, consultant and/or lectured for Boehringer Ingelheim Animal Health, Ceva, Dechra, Virbac and Zoetis. E.B. acted as a consultant or received support for studies or lectures from Boehringer Ingelheim, Elanco, MSD Animal Health, Nextmune, Vetoquinol and Virbac. T.N. received consultancy fees, lecture fees, research funding and/or other support from Aesculight, Ceva Animal Health, Elanco, Krka Animal Health, Merck, Virbac Animal Health and Zoetis. D.S. has been an investigator, consultant, lecturer and/or advisory board member for Boehringer Ingelheim Animal Health, Ceva Animal Health, Dechra, Ecuphar, Elanco, ElleVet, Hill's, Nextmune, Purina and Virbac.

## Supporting information


**Table S1:** Summary of evidence (selected studies)—topical skin barrier support.
**Table S2:** Summary of evidence—Microbiome modulation.
**Table S3:** Summary of evidence—Allergen immunotherapy.
**Table S4:** Summary of evidence—Antihistamines.
**Table S5:** Summary of evidence—Topical glucocorticoids.
**Table S6:** Summary of evidence (selected studies)—Systemic glucocorticoids.
**Table S7:** Summary of evidence—Janus kinase inhibitors.
**Table S8:** Summary of evidence—Lokivetmab.
**Table S9:** Summary of evidence—Ciclosporin.

## Data Availability

Data sharing not applicable to this article as no datasets were generated or analysed during the current study.
