# Supplementary material for: Treatment of Canine Atopic Dermatitis: Updated Guidelines From the International Committee on Allergic Diseases of Animals (ICADA)
Source: Vet Dermatol. 2026 Sep 28;37(6):963–89. doi: 10.1111/vde.70128 (PMC13751063; doi:10.1111/vde.70128)
Supplement: Supplementary file 1 [file VDE-37-963-s001.docx]

**Supplementary table 1:** Summary of evidence (selected studies) – topical skin barrier support

| ***Outcome*** | ***No. of studies*** | ***Strength of evidence*** | ***Findings / Effect*** | ***Comments*** |
| --- | --- | --- | --- | --- |
| Reduction  in pruritus | 1 RCT^26^ | B | Significant improvement in reduction of pruritic scores versus placebo; anti-pruritic effect in mild AD patients may be stronger versus moderate-to-severe AD | As complete remission was not achieved in the vast majority of dogs, it seems most useful as an adjunctive therapy in this disease. |
| Improvement  in skin lesions | 1 RCT^26^ | B | Significant improvement in reduction of lesional scores versus placebo | As complete remission was not achieved in the vast majority of dogs, it seems most useful as an adjunctive therapy in this disease. |
| Prevention  of flares (prophylaxis) | 1 RCT^28^ | C | IL-31 monoclonal antibody therapy in combination with bathing followed by a spot-on containing topical essential fatty acids, ceramides and plant extracts extended time to AD flare compared to IL-31 monoclonal antibody monotherapy | Lacks any type of topical treatment control in lokivetmab monotherapy group (uncontrolled trial) |
| Owner‑ reported satisfaction | 1 RCT^28^ | C | Significant improvement in owner global evaluation of efﬁcacy (OGATE) for IL-31 monoclonal antibody therapy in combination with bathing followed by a topical essential fatty acids, ceramides and plant extracts spot-on | Lacks any type of topical treatment control in lokivetmab monotherapy group (uncontrolled trial) |

RCT = randomised controlled trial; AD = atopic dermatitis

**Supplementary table 2:** Summary of evidence – microbiome modulation

| ***Outcome*** | ***No. of studies*** | ***Strength of Evidence*** | ***Findings / Effect*** | ***Comments*** |
| --- | --- | --- | --- | --- |
| Reduction in pruritus | 2 open-label trials^58,59^ | C | Significant reduction in pruritus compared to placebo. | Limited and inconsistent efficacy; possible placebo effect. |
| Improvement in skin lesions | 2 open-label  trials,^58,59^  1 RCT^57^ | B | Significant reduction in clinical score compared to placebo. | Improvement noted in non-treated areas as well and persisted after discontinuation of the therapy. |
| Prevention of flares (prophylaxis) | 1 RCT^53^ | C | Pre- and post-natal exposure to the probiotic *Lactobacillus rhamnosus* strain GG was associated with delay in allergen sensitization. | Possible prevention of the atopic disease. |
| Owner‑reported satisfaction | 2 open-label trials^58,59^ | C | Most owners reported high satisfaction with the treatment. | Reflects individual variability and potential placebo influence. |

RCT = randomised controlled trial

**Supplementary table 3:** Summary of evidence – Allergen immunotherapy

| ***Outcome*** | ***No. of studies*** | ***Strength of Evidence*** | ***Findings / Effect*** | ***Comments*** |
| --- | --- | --- | --- | --- |
| Reduction in pruritus | 3 RCTs^103,104,106^,  1 observational study^108^ | A | Decrease in pruritus with SCIT and ILIT, but not SLIT ^106^ | Subgroups very small ^106^, RCTs compare different types of AIT and are not placebo-controlled |
| Improvement in skin lesions | 2 RCTs^103,106^,  1 observational study^108^ | A | Decrease in lesion scores with SCIT and ILIT, but not SLIT ^106^ | Subgroups very small ^106^, RCTs compare different types of AIT and are not placebo-controlled |
| Reduction in medication scores | 2 RCTs^103,106^,  3 observational studies^100,108,207^ | A | Decrease in medication scores with SCIT, but not ILIT and SLIT ^106^ | Subgroups very small ^106^, RCTs compare different types of AIT and are not placebo-controlled |
| Owner‑reported satisfaction/quality of life scores | Many observational studies^84,93,94,99,108^ | C | In general, more than half of the owners were satisfied; subjective and variable. Improved QoL ^108^. | Unclear definition of outcome measures in many studies, different protocols and follow-up times. |

RCT = randomised controlled trial; AIT = allergen immunotherapy; SCIT = subcutaneous immunotherapy; ILIT = intralymphatic immunotherapy; SLIT = sublingual immunotherapy

**Supplementary table 4:** Summary of evidence – Antihistamines

| ***Outcome*** | ***No. of studies*** | ***Strength of Evidence*** | ***Findings / Effect*** | ***Comments*** |
| --- | --- | --- | --- | --- |
| Reduction in pruritus | 3 RCTs^114,119,120^, 1 observational study^95^ | A | Minor to absent reduction in pruritus compared to placebo; 20–30% of dogs perceived as improved. | Limited and inconsistent efficacy; possible placebo or sedative effect. |
| Improvement in skin lesions | 3 RCTs^114,119,208^ | A | No significant improvement versus placebo; fexofenadine + vitamin E modestly superior. | Heterogeneous results; unclear dose–response relationship. |
| Prevention of flares (prophylaxis) | 1 experimental study^113^ | C | Hydroxyzine failed to prevent lesion development after allergen challenge. | Unlikely to prevent acute flares. |
| Owner‑reported satisfaction | 1 survey^95^ | C | Almost 20% owners reported high satisfaction, almost 40% were somewhat satisfied; subjective and variable. | Reflects individual variability and potential placebo influence. |

RCT = randomised controlled trial

**Supplementary table 5:** Summary of evidence – Topical glucocorticoids

| ***Outcome*** | ***No. of studies*** | ***Strength of Evidence*** | ***Findings / Effect*** | ***Comments*** |
| --- | --- | --- | --- | --- |
| Reduction in pruritus and inflammation | 4 RCTs^123-125,128^, 2 open studies^126,137^ in AD; 1 RCT in ECOE*^129^ | A | Significant reduction in pruritus and skin lesions; significantly improved response to oclacitinib; improved TEWL | Quick onset of action; hydrocortisone aceponate and triamcinolone acetonide are superior to placebo; hydrocortisone aceponate is comparable to ciclosporin |
| Improvement in skin lesions | 3 RCTs^123-125^ & 2 open studies^126,137^; observational reports | A | Significant improvement in skin lesions; improved TEWL | Time to resolution dependent on type (acute vs. chronic), extent & severity of lesions; efficacy of hydrocortisone aceponate may be limited in severe chronic lesions with lichenification |
| Prevention of flares (prophylaxis) | 1 RCT in AD^127^; 2 open studies in ECOE^130,131^ | B | Long term proactive treatment on 2 days/week prevented flares for a median of 115 days (AD) and 12 months (ECOE) |  |
| Owner‑reported satisfaction | Above RCTs and open studies; numerous observational studies | A | >90% of clinicians & owners satisfied with short-term reactive & long-term proactive use |  |

RCT = randomized controlled trial; AD = atopic dermatitis; ECOE = erythroceruminous otitis externa; TEWL = transepidermal water loss

**Supplementary table 6:** Summary of evidence (selected studies) – Systemic glucocorticoids

| ***Outcome*** | ***No. of studies*** | ***Strength of Evidence*** | ***Findings / Effect*** | ***Comments*** |
| --- | --- | --- | --- | --- |
| Reduction in pruritus | 7 RCTs^121,183,208-213^ | A | Depending on the study, 50% or greater reduction in pruritus scores was generally observed between 42% to 86% of patients | Differences in studies between the type of oral systemic glucocorticoid and protocol used (frequency of administration and duration); no comparative studies between different oral glucocorticoid types and protocols. |
| Improvement in skin lesions | 7 RCTs^121,183,208-213^ | A | Depending on the study, 50% or greater reduction in pruritus scores was generally observed between 50% to 86% of patients | Differences in studies between the type of oral systemic glucocorticoid and protocol used (frequency of administration and duration); no comparative studies between different oral glucocorticoid types and protocols. |
| Prevention of flares (prophylaxis) | None | N/A | N/A | N/A |
| Owner‑reported satisfaction | 1 survey^210^ | C | Pet owners reported general satisfaction with oral prednisone/prednisolone, scoring a total of 3.87/5 (33 dogs) in satisfaction scores at day 14 of therapy | The reasoning (diseases) for the oral glucocorticoid therapy was not provided; a large range of daily glucocorticoid dosages (0.36 mg/kg to 2.64 mg/kg) was used in the study, which can influence side effects and affect overall satisfaction results |

RCT = randomised controlled trial

**Supplementary table 7:** Summary of evidence – Janus kinase inhibitors

| ***Outcome*** | ***No. of studies*** | ***Strength of Evidence*** | ***Findings / Effect*** | ***Comments*** |
| --- | --- | --- | --- | --- |
| Reduction in pruritus | Oclacitinib: 4 RCTs ^141,149-151^  Ilunocitinib:  3 RTCs^155-157^  Atinvicitinib:  2 RCTs ^163,164^ | A  A   No papers published, only abstracts | Significant and fast reduction in pruritus compared to placebo.  Superior anti-pruritic effect of ilunocitinib on oclacitinib when used every 24 hours. | Oclacitinib is comparable to ciclosporin and lokivetmab.  No published evidence for atinvicitinib. |
| Improvement in skin lesions | Oclacitinib: 4 RCTs^141,149-151^  Ilunocitinib:  3 RTCs^155-157^  Atinvicitinib:  2 RCTs ^163,164^ | A  A  No papers published as of yet, only abstracts | Significant and fast reduction in clinical score compared to placebo.  Superior anti-pruritic effect of ilunocitinib on oclacitinib when used every 24 hours. | Oclacitinib is comparable to ciclosporin and lokivetmab.    No published evidence for atinvicitinib. |
| Prevention of flares (prophylaxis) | No evidence | None |  |  |
| Owner‑reported satisfaction | No evidence | None |  |  |

RCT = randomised controlled trial

**Supplementary table 8:** Summary of evidence – Lokivetmab

| ***Outcome*** | ***No. of studies*** | ***Strength of Evidence*** | ***Findings / Effect*** | ***Comments*** |
| --- | --- | --- | --- | --- |
| Reduction in pruritus | 3 RCTs^166,177,178^, numerous observational studies | A | Significant reduction in pruritus | Quick onset of action, lokivetmab is comparable to oclacitinib and ciclosporin |
| Improvement in skin lesions | 3 RCTs^166,177,178^, numerous observational studies | A | The degree and duration of response increase with the dose | Seems more antipruritic than anti-inflammatory |
| Prevention of flares (prophylaxis) | Observational studies^170,175,176,179^ | C | Significant effect on long term use | Association with topical treatments may extend time to flare ^28,170^ |
| Owner‑reported satisfaction | Numerous observational studies | C | Up to 93% of owners satisfied with long term use |  |

RCT = randomised controlled trial

**Supplementary table 9:** Summary of evidence – Ciclosporin

| ***Outcome*** | ***No. of studies*** | ***Strength of Evidence*** | ***Findings / Effect*** | ***Comments*** |
| --- | --- | --- | --- | --- |
| Reduction in pruritus | Numerous^1^ | A | Significant reduction in pruritus compared to placebo. Equivalent reduction compared to prednisolone, lokivetmab,^178^ hydrocortisone aceponate^125^ and oclacitinib.^151^ | Slow onset of action requiring the co-administration of other immunomodulatory treatments.^143^ |
| Improvement in skin lesions | Numerous^1^ | A | Significant reduction in clinical score compared to placebo. Equivalent reduction compared to prednisolone, hydrocortisone aceponate^125^ and oclacitinib.^151^ | Slow onset of action requiring the co-administration of other immunomodulatory treatments.^143^ |
| Prevention of flares (prophylaxis) | No evidence | None |  |  |
| Owner‑reported satisfaction | No evidence | None |  |  |

RCT = randomised controlled trial
